# Photocatalytic Vanadium‐Mediated Amination of Benzene With Hydroxylamine

**DOI:** 10.1002/chem.202503475

**Published:** 2026-03-28

**Authors:** Dana Králová, Maximilian Philipp, Matthias Schmalzbauer, Thilo Reiter, Bernd Schäfer, Ruth M. Gschwind, Burkhard König

**Affiliations:** ^1^ Fakultät Für Chemie und Pharmazie Universität Regensburg Regensburg Germany; ^2^ BASF SE Ludwigshafen am Rhein Germany

**Keywords:** ^51^V‐NMR, aniline synthesis, kinetic analysis, photocatalysis, vanadium catalysis

## Abstract

The synthesis of aromatic amines, especially anilines, is crucial in organic chemistry because of their significance in pharmaceuticals and high‐value chemicals. However, traditional methods for producing aniline involve pre‐functionalization and/or several high‐energy‐demanding steps. Therefore, the development of direct protocols towards aniline has attracted significant interest. We introduce a direct photocatalytic method for synthesizing aniline using hydroxylamine and vanadium complexes. While previous studies describe vanadium complexes and hydroxylamine in thermally driven reactions, our research utilizes photochemistry, providing milder reaction conditions for the efficient benzene amination. Mechanistic investigations using ^51^V‐NMR support coordination of hydroxylamine to vanadium and the multifaceted role of acetic acid as solvent, acid, and ligand. X‐band EPR and ^51^V‐NMR demonstrated the time‐dependent reduction of vanadium by hydroxylamine. The same excess experiments and ^15^N‐NMR coordination studies identified product inhibition by aniline. ^1^H‐NMR kinetic experiments, enabled through a broad peak suppression, suggest a paramagnetic V^+4^‐hydroxylamine complex as the key intermediate, and ^51^V‐NMR kinetics support the photocatalyst's role in maintaining the redox cycle and reducing the active V^+4^‐hydroxylamine intermediate. Our direct photocatalytic method not only enables the selective synthesis of aniline under mild conditions, but also allows easy scaling through its compatibility with flow systems.

## Graphical abstract

Photochemical activation of *N*‐phenylphenothiazine (PPT) enables a selective direct benzene amination with hydroxylamine in the presence of various vanadium catalysts under mild reaction conditions. Spectroscopic and kinetic analyses identify a key vanadium(IV)‐hydroxylamine intermediate, elucidate the crucial roles of the acidic medium, the photocatalyst, and the solvent, and demonstrate inhibition by aniline.

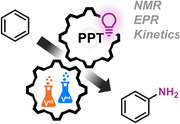

## 1 Introduction

The synthesis of aromatic amines, particularly anilines, holds significant importance in organic synthesis owing to the ubiquity of nitrogen‐containing functional groups in pharmaceuticals [1] and various high‐value chemicals [2]. Aniline, the simplest aromatic amine, serves as a key precursor for dyes, pharmaceuticals, pesticides, polymers, and other industrially relevant chemicals [3], with a global consumption reaching 10.4 million tonnes in 2024 and continuing to rise [4]. Given the importance mentioned above, several synthetic routes to primary anilines have been developed [5]. The very successful methods, including nitration of arenes followed by reduction of the nitroarene intermediates [6] or Ullmann [7, 8]/Chan‐Lam [9]/Buchwald‐Hartwig [10, 11] type C─N cross‐couplings, require the pre‐functionalization (nitration, halogenation, or borylation) step. Moreover, the nitro‐reduction predominantly used in industrial production consumes significant energy, requires a stoichiometric amount of reducing agent, and generates substantial waste. Therefore, the direct amination of benzene has attracted considerable attention as a more atom‐ and energy‐efficient, environmentally sustainable alternative.

**SCHEME 1:**
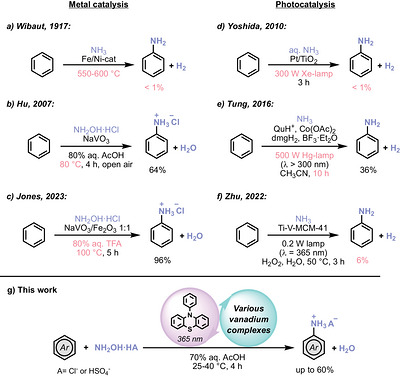
Examples of the developed direct approaches towards anilines utilizing metal catalysis (a–c) and photocatalysis (d–f), and our dual catalytic approach (g). The disadvantages of the current protocols are highlighted in pink.

The first direct synthesis of aniline from benzene was reported by Wibaut in 1917 via reaction of benzene with ammonia over Fe or Ni catalysts at 550°C–600°C (Scheme 1a). [12] Subsequent studies explored alternative metal catalysts (W, Mo, Zr, etc.) to improve yields, but these methods remained limited by high energy demand and low benzene conversion [13, 14, 15, 16]. Besides this approach, several other methods have been developed to obtain aniline directly from benzene. Other strategies for direct amination include acid‐catalysed electrophilic amination with azides (e.g., TMSN_3_/TfOH or NaN_3_/BF_3_‐H_2_O). [17, 18, 19, 20, 21] Although these early protocols showed poor atom efficiency, Kappe and co‐workers later optimized the process in continuous flow, achieving an 86% yield of aniline under safer and more efficient conditions [21].

Direct benzene amination has also been investigated in heterogeneous catalysis employing modified zeolites (MCM‐41 [22, 23, 24, 25], AlPO_4_‐5 [26], TS‐1 [27]), graphene oxide [28, 29], and Cu(II)‐nanoclusters [30] for amination by hydroxylamine. Among these, only Ni‐doped AlPO_4_‐5 proved efficient, providing aniline in 73% yield. Homogeneous catalysis and electrochemical methods with hydroxylamine derivatives have been explored as well [31, 32, 33, 34, 35, 36, 37, 38, 39, 40, 41]. In particular, vanadium catalysts have attracted extensive attention and were able to generate up to 96 mol% of aniline (Scheme 1b,c) [36, 37, 38, 39, 40]. However, these methods required temperatures  ≥ 80°C and, in the case of the protocol reported by Jones, also highly corrosive and environmentally persistent trifluoroacetic acid (TFA) [42] as solvent.

Additionally, strategies involving light have also been reported. In 1991, Adachi et al. generated nitrenium ions from 1‐aminopyridinium and 1‐aminoquinolinium salts under 75 W Hg‐lamp irradiation [43]. Yoshida et al. later employed Pt‐loaded TiO_2_ with Xe‐lamp irradiation for photocatalytic amination by ammonia, though yields did not exceed 1% (Scheme 1d) [44, 45]. More recently, Tung et al. achieved 40% benzene conversion with 90% selectivity using ammonia and a dual quinolinium‐cobalt catalytic system under Hg‐lamp irradiation (Scheme 1e) [46]. In 2022, Zhu et al. reported heterogeneous photocatalysis with Ti‐ and V‐loaded MCM‐41, producing up to 6% aniline from benzene and ammonia under UV irradiation at 50°C (Scheme 1f) [47].

Mechanistic investigations have also been carried out on vanadium‐catalyzed one‐step benzene amination, with particular emphasis on sodium metavanadate (NaVO_3_) and hydroxylamine interactions [37]. Zhu et al. applied a multi‐spectroscopic approach, including nuclear magnetic resonance of the diamagnetic V^+5^ species (^51^V‐NMR), electron paramagnetic resonance (EPR) of paramagnetic V^+4^ complexes, and ultraviolet‐visible absorption spectroscopy (UV‐Vis), to elucidate the structure of vanadium species and the reaction mechanism of their thermal approach. Their analysis indicated coordination of hydroxylamine, water, and acetic acid with pentavalent vanadium, while pH‐dependent vanadium chemical shifts revealed changes in speciation under neutral or basic pH [48, 49, 50].

Herein, we report a one‐step arene amination combining metal‐ and photocatalysis. Catalytically efficient, inexpensive, and earth‐abundant vanadium complexes are employed together with light as the energy source, enabling milder reaction conditions. Using *N*‐phenylphenothiazine (PPT) as a photocatalyst, anilines were obtained in fair to good yields at ambient or slightly elevated temperatures. Complementary spectroscopic studies further elucidated the interactions of the reaction components with the vanadium species.

## 2 Results and Discussion

### 2.1 Optimization, Metal Catalysts, and Substrate Scope

**FIGURE 1:**
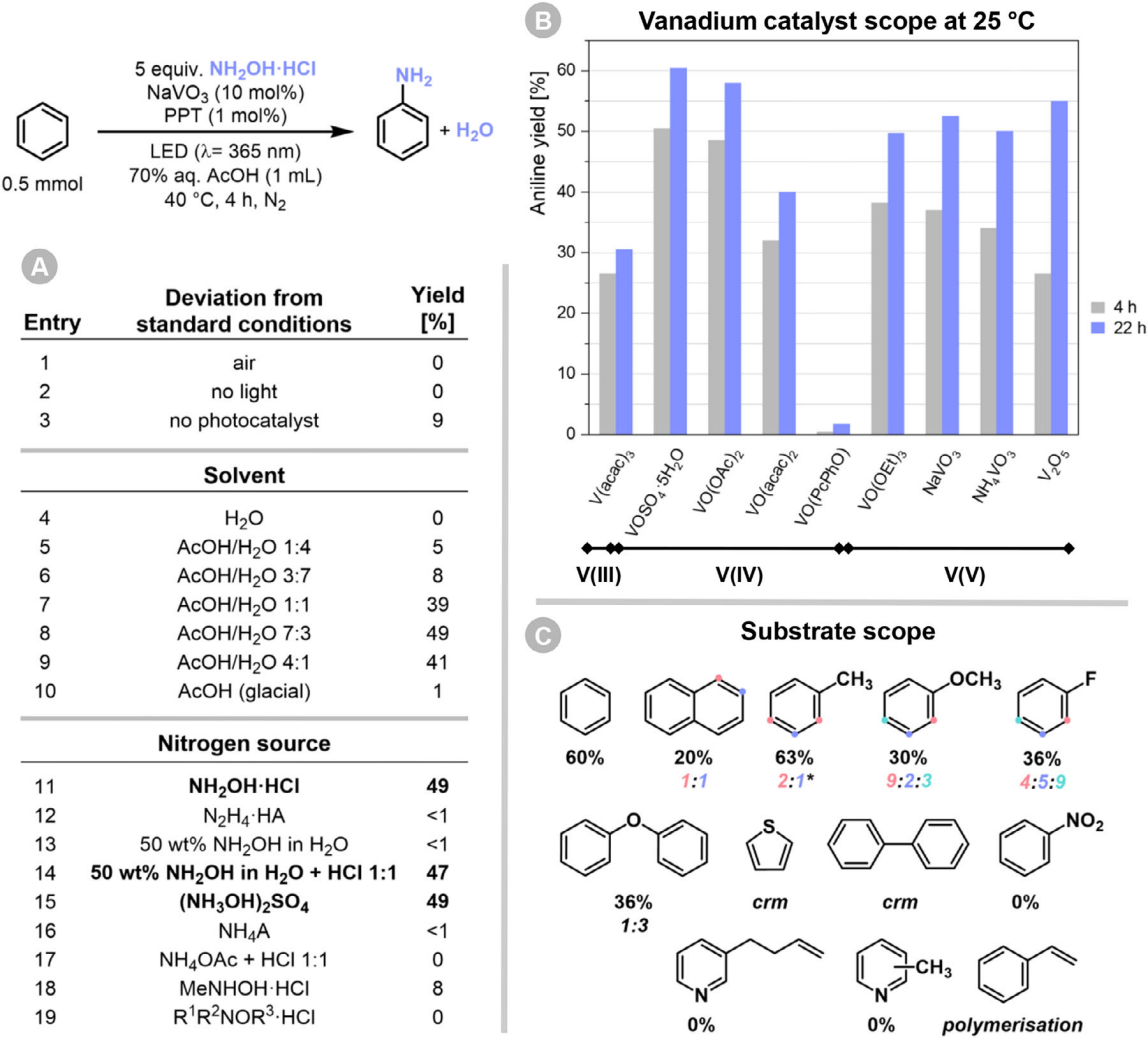
Optimizations of benzene amination and substrate scope. Yields were determined by GC‐FID with toluene as internal standard. (A) Controls, optimization of solvent ratio, and nitrogen sources for benzene amination. A = AcO^−^ or Cl^−^; R^1^ = H or Et; R^2^ = H, Et, Me, Ac or Bz; R^3^ = H, Me or p‐NO_2_‐Bz. (B) Scope of vanadium catalysts for benzene amination by hydroxylamine. Reactions were conducted at 25°C instead of 40°C. (C) Substrate scope and limitations of the aromatic compounds for amination. VO(OAc)_2_ was used as a metal catalyst. *o‐ and p‐toluidines were inseparable on GC‐FID; crm = complex reaction mixture.

While the thermal vanadium‐mediated aminations [36, 37, 38, 39, 40] of benzene represent an important contribution, the requirement for elevated temperatures and environmentally persistent solvents limits broader applicability. We therefore investigated the combination of vanadium catalysis with photocatalysis to modulate the reaction pathways under non‐thermal conditions. Our investigation started with optimization of the photocatalytic reaction conditions for benzene amination by hydroxylamine (Figure 1A). Using glacial acetic acid led to poor solubility of hydroxylamine hydrochloride and aniline yields of only ∼1% (**entry 10**). Acetic acid was, however, essential for efficient conversion, as other acids or the replacement of water with an organic solvent decreased the yield (Tables S3–S5). The optimal result was obtained with a 70% aqueous acetic solution (**entry 8**).

Different nitrogen sources were subsequently screened for the benzene amination (**entries 11–19**, more detailed in Table S6). Hydrazine salts gave negligible yields (**entry 12**), likely due to the higher N─N bond dissociation energy [51] compared with the N─O bond in hydroxylamine [52]. Interestingly, using a 50 wt% hydroxylamine solution instead of its hydrochloride salt resulted in  < 1% yield, highlighting the pH dependency of the reaction (**entry 13**). In situ formation of hydroxylamine hydrochloride by the addition of HCl restored the yield (**entries 11** and **14**), and hydroxylammonium sulfate provided similar results (**entry 15**). Ammonium salts failed to produce aniline even after the addition of HCl (**entries 16** and **17**). *N*‐Methyl hydroxylamine hydrochloride afforded only a low yield (**entry 18**), and other *N*‐ and *O*‐alkylated or acylated hydroxylamine derivatives were unreactive under the given conditions (**entry 19**), suggesting that any substitution on oxygen inhibits the coordination to vanadium.

To explore the effect of the initial oxidation states as well as the ligands and counter ions of the vanadium catalyst complex used, several different vanadium catalysts were evaluated at room temperature (Figure 1B). Interestingly, no direct correlation between vanadium oxidation state and yield was observed, hinting at an interconversion of the different oxidation states during the reaction, while the solubility of the vanadium complex emerged as a critical factor. Literature reports [38] further indicate that the electron density at vanadium (its electrophilicity) and the steric effects of ligands significantly affect the degree of amination. In addition to the vanadium‐catalyst variability, the benzene amination by hydroxylamine provided aniline exclusively, with no by‐products observed (Figure S30). Polyamination and oxidation of the formed aniline are suppressed due to the inert atmosphere and protonation of the nitrogen in an acidic medium.

Amination of other aromatic substrates proved challenging due to their low solubility in the reaction mixture (Figure 1C). Arenes bearing electron‐donating groups afforded the corresponding aminated products to some extent, with only toluene providing toluidines in an overall yield comparable to aniline. Electron‐deficient substrates, such as nitrobenzene and pyridine derivatives, remained unreactive, while styrene polymerised under the reaction conditions. The observed substitution pattern represents an intermediate case between a non‐selective radical pathway and an electrophilic aromatic substitution with pronounced *ortho/para* selectivity. Scale‐up to 1.5 mmol in a microflow reactor (Figure S4) using VO(acac)_2_ afforded 19% aniline after one hour of irradiation, matching the batch yield.

### 2.2 Coordination of Hydroxylamine to the Vanadium Catalysts Investigated by ^51^V‐NMR and EPR Spectroscopy

**FIGURE 2:**
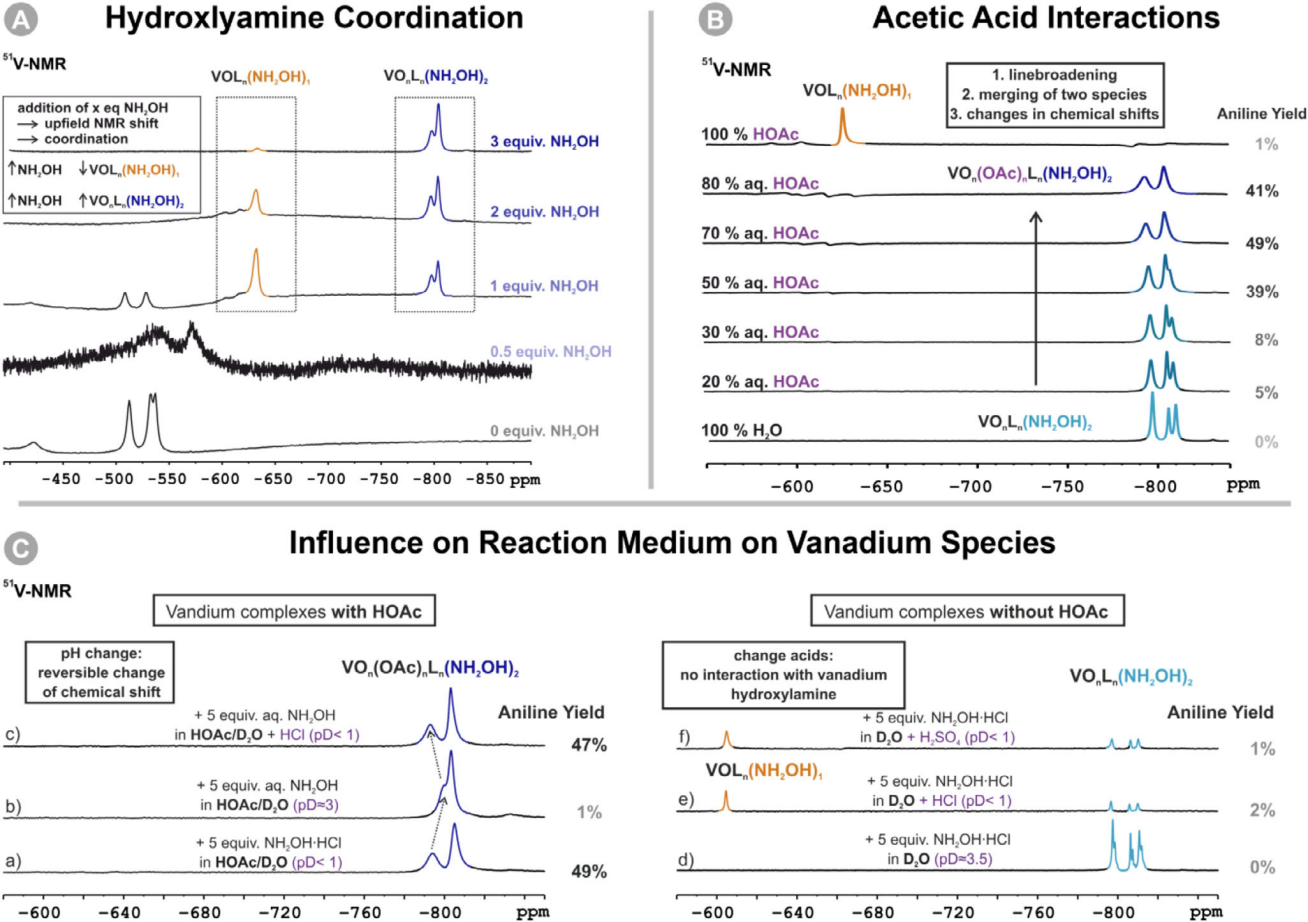
(A) ^51^V‐NMR spectra (normalized to the maximum peak) of NaVO_3_ (0.05 M) with varying amounts of NH_2_OH∙HCl in HOAc/D_2_O (7:3). At low hydroxylamine loadings, broad resonances between −500 and −700 ppm are observed, consistent with fast‐exchanging vanadium complexes. Upon addition of ∼1 equiv. of hydroxylamine, a dominant signal appears at approx. −632 ppm, while further increase to  ≥ 2 equiv. leads to suppression of the original vanadate signals and predominance of resonances at −794 and −804 ppm, consistent with sequential formation of first mono‐ and then bis‐hydroxylamine vanadium adducts and saturation of the coordination. (B) ^51^V‐NMR of NaVO_3_ (0.05 M) with 2.5 M NH_2_OH∙HCl in varying ratios of HOAc/H_2_O (X:X). Increasing acetic acid content shifts vanadium speciation: in pure water, narrower resonances between −790 and −820 ppm correspond to bis‐hydroxylamine species with no activity; at 50%–70% HOAc, signals merge at −804 ppm, coinciding with enhanced catalysis; in glacial acetic acid, the spectrum is dominated by a mono‐hydroxylamine species, reflecting limited hydroxylamine solubility and ligand availability. (C) ^51^V‐NMR spectra of NaVO_3_ (0.05 M) with different acids and pD values. 5 equiv. NH_2_OH∙HCl (2.5 M) is with respect to 0.5 M benzene at reaction conditions. (a) Characteristic vanadium shifts of two hydroxylamine molecules are observed, and the reaction is successfully proceeding. (b) Increasing the pD with aq. NH_2_OH induces a downfield shift of the left vanadium peak, indicating a pD/pH sensitivity. Under these conditions, aniline formation is largely suppressed. (c) Re‐acidification with HCl restores the shifts and catalytic activity, highlighting the crucial role of the corresponding pH/pD‐dependency of this catalytic system. (d) Only water as a solvent stops the reaction and forms different complexes within the same chemical shift range but with narrower linewidths. This demonstrates that acetic acid is involved in the vanadium complex. (e) In HCl medium, most vanadium species remain coordinated to only one hydroxylamine ligand. (f) H_2_SO_4_ leads to the same results as with HCl, with no exchange‐broadened peaks, and most of the species in the complex have only one hydroxylamine ligand. L_n_: H_2_O, HOAc, oxide.

Since the synthetic screening identified hydroxylamine as a crucial nitrogen source, spectroscopic investigations of diamagnetic vanadium species were conducted to elucidate the interaction between NH_2_OH and the vanadium catalyst. Literature reports describe vanadium complexes bearing one or two coordinated hydroxylamine ligands [37, 48]. To probe the formation of analogous species, ^51^V‐NMR spectroscopy was employed, as the chemical shift is highly sensitive to changes in the vanadium coordination environment and ligand binding. A gradual addition of hydroxylamine up to three equivalents relative to the diamagnetic NaVO_3_ (0.05 M) in 70% aqueous acetic acid induced pronounced upfield shifts (Figure 2A), indicating substantial changes in the vanadium environment upon ligand interaction.

NaVO_3_ in 70% aq. AcOH exhibited multiple ^51^V‐NMR resonances around −500 ppm (Figure 2A), consistent with a distribution of vanadate species, including VO_2_^+^ and mono‐ and polyoxidovanadates [53, 54, 55], possibly involving acetate coordination. Upon addition of 0.5 equivalents of hydroxylamine, very broad signals appeared at approximately −500, −575, and −700 ppm. These features likely arose from a dynamically exchanging mixture with a medium exchange rate on the NMR timescale of heterocomplexes involving water, acetate, and hydroxylamine as potential ligands. Furthermore, the overall line broadening is consistent with the quadrupolar nature of the ^51^ V nucleus and its pronounced sensitivity [56]. Increasing the hydroxylamine loading to one equivalent led to sharpening of the resonances and the emergence of new signals at −632, −794, and −804 ppm. The dominant species was observed at −632 ppm, accompanied by minor components at −794 and −804 ppm, while residual signals from vanadate species lacking hydroxylamine coordination remained detectable (signals around −500 ppm). Upon addition of two equivalents of hydroxylamine, the initial vanadate resonances are largely suppressed, and the spectral population is shifted predominantly toward the signals near −800 ppm. Further titration to 3 equivalents reinforced this trend, with the −632 ppm signal becoming minor and the −794/−804 ppm signals dominating the spectrum. No major changes in chemical shift or relative intensities have been observed upon addition of excess hydroxylamine, indicating saturation behaviour with two hydroxylamine ligands.

The systematic and saturating evolution of the ^51^V‐NMR spectra strongly supports direct interaction of hydroxylamine with vanadium as a ligand under the applied conditions. The appearance of two distinct spectral regimes is consistent with the formation of mono‐ and bis‐hydroxylamine adducts, in agreement with previous reports [37, 48]. Crystal structures for similar V(V)‐bis‐hydroxylamine complexes showed comparable chemical‐shift regions in ^51^V‐NMR [57], further corroborating the coordination of two hydroxylamine molecules. While unambiguous structural or specific oxidation‐state assignments cannot be made based solely on the presented data, again, the observed trends provide strong evidence for substantial modification of the vanadium coordination environment upon hydroxylamine addition. The coordination sphere likely additionally involves solvent‐derived ligands such as water or acetate, which is investigated next.

In addition to hydroxylamine as the amination source, acetic acid exerted a pronounced influence on the catalytic activity. To elucidate its role, ^51^V‐NMR titration experiments of vanadium‐hydroxylamine systems were performed with increasing concentration of acetic acid (Figure 2B). In pure water, three main ^51^V‐NMR signals between −790 and −820 ppm with relatively narrow line widths were observed, in agreement with prior observations attributed to multiple vanadium species bearing two hydroxylamine ligands. Under these conditions, no catalytic activity was detected. Increasing the fraction of AcOH induces progressive line broadening, systematic chemical shift changes, and a simultaneous increase in the reaction yield. At intermediate AcOH concentrations (50%–70%), two previously resolved resonances at −806 and −810 ppm merge into a single signal at approximately −804 ppm, alongside a significant enhancement in catalytic performance. In contrast, predominantly a signal attributed to a mono‐hydroxylamine vanadium species was observed in glacial acetic acid, with almost no productive reaction occurring. This reflects the limited solubility of hydroxylamine, resulting in reduced ligand availability and highlights again the necessity of having two hydroxylamine ligands bound for a productive reaction. Collectively, these titration studies support that acetic acid plays, next to the coordination of hydroxylamine, a critical role in modulating vanadium speciation, coordination geometries and catalytic performance, consistent with its involvement as a labile ligand or altering the dynamics of these vanadium hydroxylamine complexes.

To disentangle the acidity effects from the specific coordination effects of AcOH, we probed how the pD value and the acid type influence the vanadium speciation and the catalytic performance. Under reaction conditions in 70% aq. AcOH, solutions containing NaVO_3_ and a large excess of hydroxylamine hydrochloride (50 equivalents, pD < 1) displayed two broad resonances at −794 and −804 ppm (Figure 2Ca), consistent with bis‐hydroxylamine vanadium species. Increasing the pD to approximately three by employing aqueous hydroxylamine instead of the hydrochloric salt resulted in a shift of the −794 ppm resonance toward −800 ppm, while the −804 ppm signal remained largely unaffected (Figures 2Cb). Alongside, catalytic activity is strongly suppressed. Re‐acidification with HCl restores both the original spectral features and the catalytic performance (Figures 2Cc). Although this correlation suggests involvement of this pD‐sensitive vanadium species in the productive pathway, acidity may also affect other components of the system, including photocatalyst stability and the lifetime or concentration of the *N*‐centred radicals. Subsequently, the influence of AcOH was tested against other acids. As previously shown, the replacement of acetic acid by water yielded narrower resonances and additional spectral features (Figures 2Cd). The same signals were also observed with strong inorganic acids (HCl and H_2_SO_4_, pD < 1; Figures 2Ce, f). Additionally, these acids favour the formation of potentially unproductive mono‐hydroxylamine vanadium species (≈ −610 ppm). Replacement of acetic acid led to almost no aniline generation. Both low pD/pH and acetic acid are therefore required for an effective reaction. Overall, these observations demonstrate that, in addition to coordinating hydroxylamine ligands, acetic acid at sufficiently low pD is key to a productive transformation. Acetic acid seems to play a complex and possibly multifaceted role as solvent, proton source, and labile ligand, thereby likely modulating coordination equilibria and exchange dynamics and promoting the formation of catalytically active vanadium‐hydroxylamine species.

Not only diamagnetic vanadium(V) complexes, such as NaVO_3_, extensively characterised in the first part, but also paramagnetic vanadium(IV) species appeared to participate in the catalytic cycle. Comparable reaction yields obtained for different catalyst precursors suggest interconversion between vanadium oxidation states under reaction conditions. Although vanadium(IV) complexes (e.g., VO(acac)_2_, VO(OAc)_2_) cannot be directly observed by ^51^V‐NMR spectroscopy due to their paramagnetic nature, indirect detection could support their involvement. ^1^H‐NMR spectra of VO(acac)_2_ under reaction conditions revealed free acetylacetonate, while GC‐MS analysis detected an isoxazole derivative (Figures S9 and S31), indicating a partial ligand exchange and coordination of hydroxylamine to vanadium(IV), analogous to the species proposed for vanadium(V).

**FIGURE 3:**
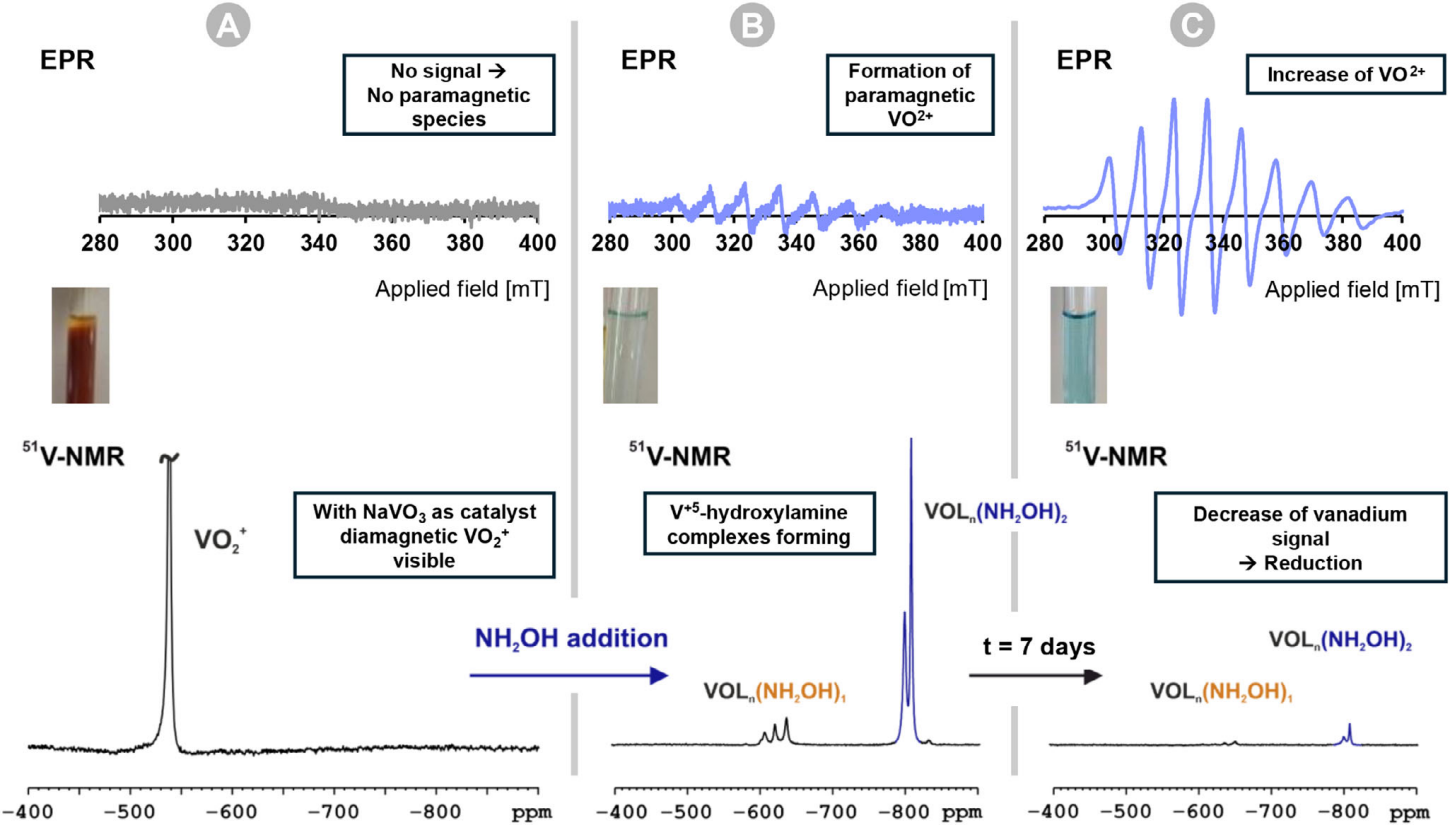
X‐band EPR and ^51^V‐NMR spectra of the vanadium catalysts in 70% aq. AcOH. (A) Without hydroxylamine, no signal is detected in the EPR, thus, no EPR‐active vanadium(IV) is present, while the single signal at ‐540 ppm attributed to VO_2_^+^ is observed in the NMR. EPR: *ν*_mw_ = 9.460306 GHz. (B) Upon addition of ^15^N‐labelled hydroxylamine, the previously shown vanadium‐hydroxylamine complexes emerged in NMR. Furthermore, a signal in the EPR appeared showing a reduction of vanadium(V) to (IV) through the reaction with hydroxylamine as a reducing agent. EPR: *ν*_mw_ = 9.458926 GHz. (C) After 7 days, a strong EPR signal and only minor amounts of V^+5^ species remaining in NMR were detected. This illustrates the time‐dependent reduction of diamagnetic V^+5^ to paramagnetic V^+4^. EPR: *ν*_mw_ = 9.460882 GHz.

To examine whether hydroxylamine induces in situ reduction of vanadium(V) to vanadium(IV) and to gain deeper mechanistic insight into the redox dynamics of the system, complementary EPR and ^51^V‐NMR spectra were recorded (Figure 3). The diamagnetic NaVO_3_ alone was EPR silent and showed a singlet at −540 ppm in the ^51^V‐NMR spectrum (Figure 3A). Upon addition of hydroxylamine hydrochloride, a weak paramagnetic signal corresponding to VO^2+^ appeared in EPR [58, 59], accompanied by vanadium(V)‐hydroxylamine complexes described above with ^51^V‐NMR (Figure 3B) and a color change to light green. After 7 days, the solution turned bright turquoise blue, and the EPR signal of vanadium(IV) species became dominant, while the ^51^V‐NMR signals of vanadium(V) complexes almost disappeared (Figure 3C). This predominantly reduced state resulted in no aniline formation, most likely either due to the formation of unproductive vanadium(IV) complexes or due to the decomposition of hydroxylamine, which prevents the generation of catalytically active vanadium(IV)‐hydroxylamine species.

Mass spectrometry was also employed to obtain additional structural insight into the vanadium complexes in the reaction mixture. However, no conclusive results were obtained.

These results show in situ reduction of vanadium(V) to vanadium(IV) by hydroxylamine under strongly acidic conditions corroborating previous reports [37]. Ligand‐exchange evidenced by free acetylacetonate (acac) in the ^1^H‐NMR after addition of hydroxylamine to VO(acac)_2_ with no vanadium(V)‐bis‐hydroxylamine present further indicates the existence of analogous vanadium‐hydroxylamine complexes for both oxidation states (Figure S9). The observed time‐dependent interconversion demonstrates that the initial vanadium oxidation state is of minor importance if conversion to the catalytically active species occurs.

### 2.3 Kinetic Insights Into Reaction Dynamics

**FIGURE 4:**
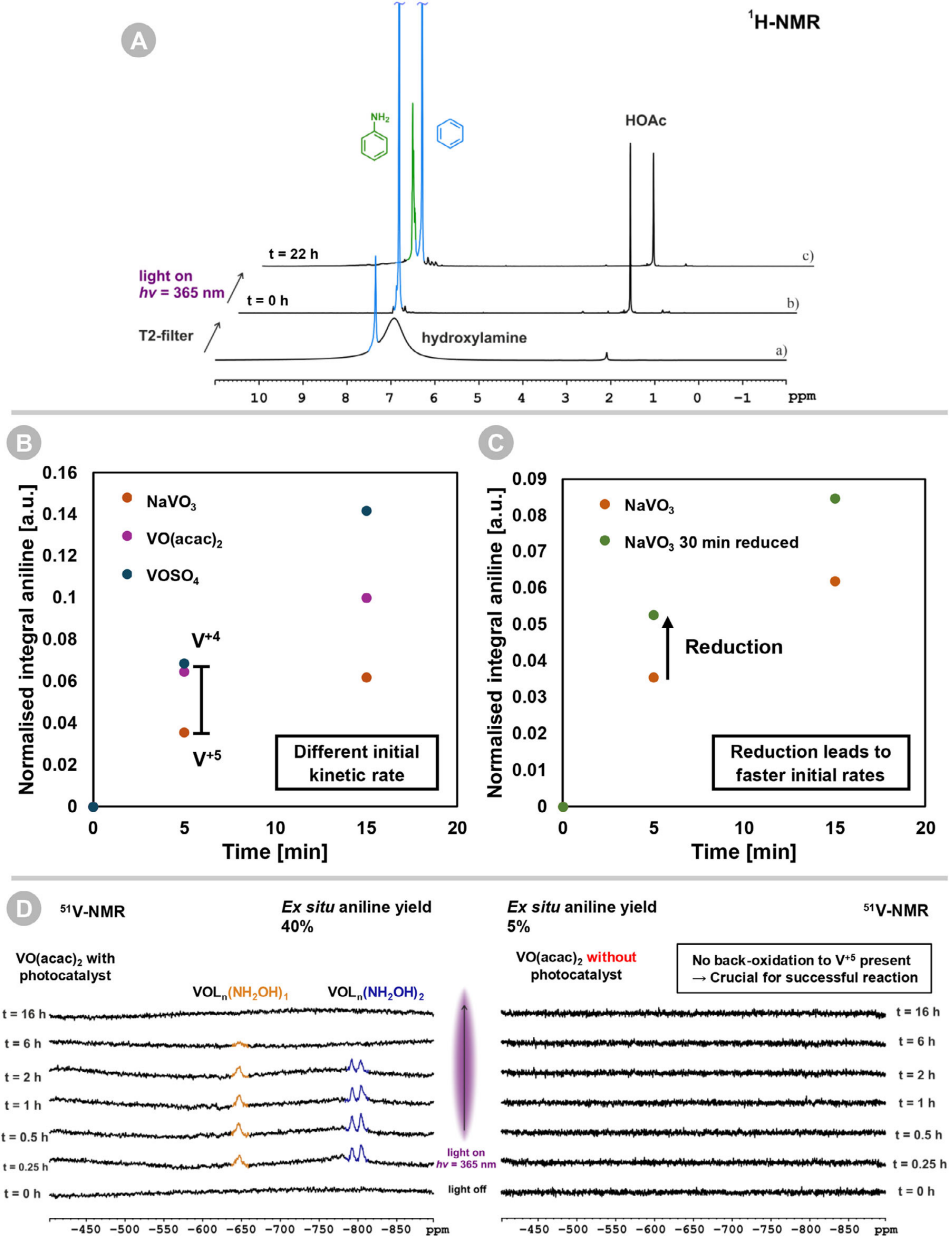
(A) ^1^H‐NMR spectra of the vanadium‐catalyzed amination of benzene (0.5 M in 70% aq. AcOD‐d_4_) with NaVO_3_ (10 mol%), PPT (2 mol%), and NH_2_OH∙HCl (5 equiv.) at 298 K. (a) Overlapping broad hydroxylamine signals made integration of benzene and aniline impossible. (b) With the PROJECT pulse sequence as a T2 filter (D2 (cycle time) = 0.001 s, L31 (loop counter) = 32), the broad disturbing peak could be removed. (c) Spectrum after 22 h of in situ irradiation with formed aniline. (B) Qualitative kinetic curves derived from ex situ ^1^H‐NMR of the photocatalytic amination of benzene with VO(acac)_2_, VOSO_4_, and NaVO_3_ showed a slower initial rate for the V^+5^ catalyst, supporting a crucial V^+4^ intermediate_._ (C) Qualitative kinetic curves derived from ex situ ^1^H‐NMR of the photocatalytic amination of benzene with NaVO_3_ and pre‐reduced NaVO_3_. Pre‐reduction by 30 min stirring in the dark led to higher initial rates, additionally favouring a V^+4^ intermediate. Reaction conditions: 0.5 mmol benzene, 5 equiv. NH_2_OH·HCl, 10 mol% V‐catalyst, 1 mol% PPT, 1 mL aq. AcOH, 298 K, LED (365 nm). (D) Comparing the VO(acac)_2_‐catalysed reaction at 298 K with and without photocatalyst, NMR spectra show no back‐oxidation and almost no product formation without PC. Reaction conditions: 0.5 mmol benzene, 5 equiv. NH_2_OH·HCl, 10 mol% V‐catalyst, 2 mol% or no PPT, 1 mL aq. AcOD‐d_4_, 298 K, in situ NMR irradiation (365 nm).

The interconversion between vanadium(IV) and (V) species offers an opportunity to identify the catalytically active oxidation state by monitoring initial reaction kinetics. However, kinetic monitoring by conventional ^1^H‐NMR proved challenging because the benzene resonance overlapped with a broad (∼600 Hz) signal from hydroxylamine in the paramagnetic environment, rendering deconvolution ineffective. By employing advanced T_2_‐weighted spectra using the PROJECT (Periodic Refocusing of J Evolution by Coherence Transfer) pulse sequence [60], the broad signal could be suppressed, thus enabling reliable kinetic evaluation with clear spectra (Figure 4A).

Under photocatalytic conditions, direct comparison of ex situ kinetics using VO(acac)_2_, NaVO_3_, and VOSO_4_ revealed that both vanadium(IV) catalysts, VOSO_4_ and VO(acac)_2_, show higher initial reaction rates than the vanadium(V) catalyst after 5 min (Figure 4B). Consistent with this trend, in situ experiments showed that the vanadium(V) catalyst (NaVO_3_) produces aniline at a lower initial rate than VO(acac)_2_ (Figure S10). The presence of photocatalyst and light under reaction conditions accelerates the redox processes, resulting in only a small initial rate offset between the systems. Nevertheless, the faster initial rates observed for vanadium(IV) catalysts indicate that vanadium(IV) complexes are the catalytically relevant intermediates. Corroborating this, initial pre‐reduction of NaVO_3_ by stirring with hydroxylamine in the dark for 30 min led to enhanced conversion during the 15 min of reaction, further supporting the assignment of vanadium(IV)‐hydroxylamine complexes as the catalytically active intermediates (Figure 4C). To our surprise, prolonged reduction (18 h) of NaVO_3_ to V^+4^ prior to the reaction resulted in overall lower activity, suggesting a formation of catalytically inactive, kinetically trapped vanadium(IV) species and/or accelerated decomposition of hydroxylamine (Figure S13). ^51^V‐NMR spectra revealed under partial reduction (30 min), bis‐hydroxylamine vanadium(V) intermediates, associated with productive aniline synthesis, were present, whereas after the prolonged reduction, these intermediates were absent, independent of the vanadium precursor (Figure S17). The apparent contradiction thus arises from two distinct regimes: (i) productive vanadium(IV)‐hydroxylamine species generated under controlled pre‐reduction, which remain redox‐accessible with vanadium(V), and (ii) over‐reduced vanadium(IV) species that cannot generate catalytically active vanadium(IV)‐hydroxylamine intermediates.

To verify that benzene amination is the dominant process under these conditions, kinetic experiments using ^15^N‐labelled hydroxylamine were conducted (Figure S19). Without light, only free hydroxylamine and its reduction products, ammonium ion and N_2_O, were observed (Figures S20). Upon illumination, aniline appeared as the sole organic nitrogen product [61], with N_2_O [62], and NH_4_^+^ formed from ongoing hydroxylamine disproportionation. This confirms that the photocatalytic amination proceeds with high selectivity.

Further in situ ^51^V‐NMR studies elucidated the behaviour of vanadium species during catalysis. For the photocatalytic reaction with NaVO_3_, concentrations of vanadium(V)‐hydroxylamine complexes decreased slightly throughout the reaction (Figure S15). In contrast, under thermal conditions (343 K), rapid reduction occurred within 30 min without reoxidation, consistent with temperature‐accelerated hydroxylamine reduction (Figure S16). As expected, both vanadium(V) and vanadium(IV) complexes were found to form the same bis‐hydroxylamine intermediates in ex situ kinetics (Figure S14), supporting convergence to a common catalytic manifold independent of the initial vanadium oxidation state. For the photocatalytic in situ reaction with VO(acac)_2_, no initial V^+5^ species were detected. Instead, vanadium was transiently re‐oxidised during the reaction to form vanadium(V)‐hydroxylamine intermediates, which were subsequently reduced again (Figure 4D, left). In addition, vanadium(V)‐mono‐hydroxylamine complexes were observed, suggesting oxidation of distinct vanadium(IV)‐mono‐hydroxylamine complexes that persist despite the large stoichiometric excess of hydroxylamine. Comparing it to the in situ irradiation without a photocatalyst at 298 K, VO(acac)_2_ showed only minor aniline formation with vanadium remaining in its reduced form (Figure 4D, right). This supports the role of the photocatalyst in sustaining redox turnover by re‐oxidising vanadium(IV)‐hydroxylamine complexes to vanadium(V)‐hydroxylamine and also accelerating the product‐generating step. Further control experiments performed in the absence of benzene, with and without the photocatalyst, again revealed pronounced accumulation of vanadium(V)‐bis‐hydroxylamine intermediates in the presence of the photocatalyst. (Figure S18). Collectively, these observations support the involvement of light and the photocatalyst in promoting reaction progression and oxidation of the vanadium(IV)‐hydroxylamine species.

**FIGURE 5:**
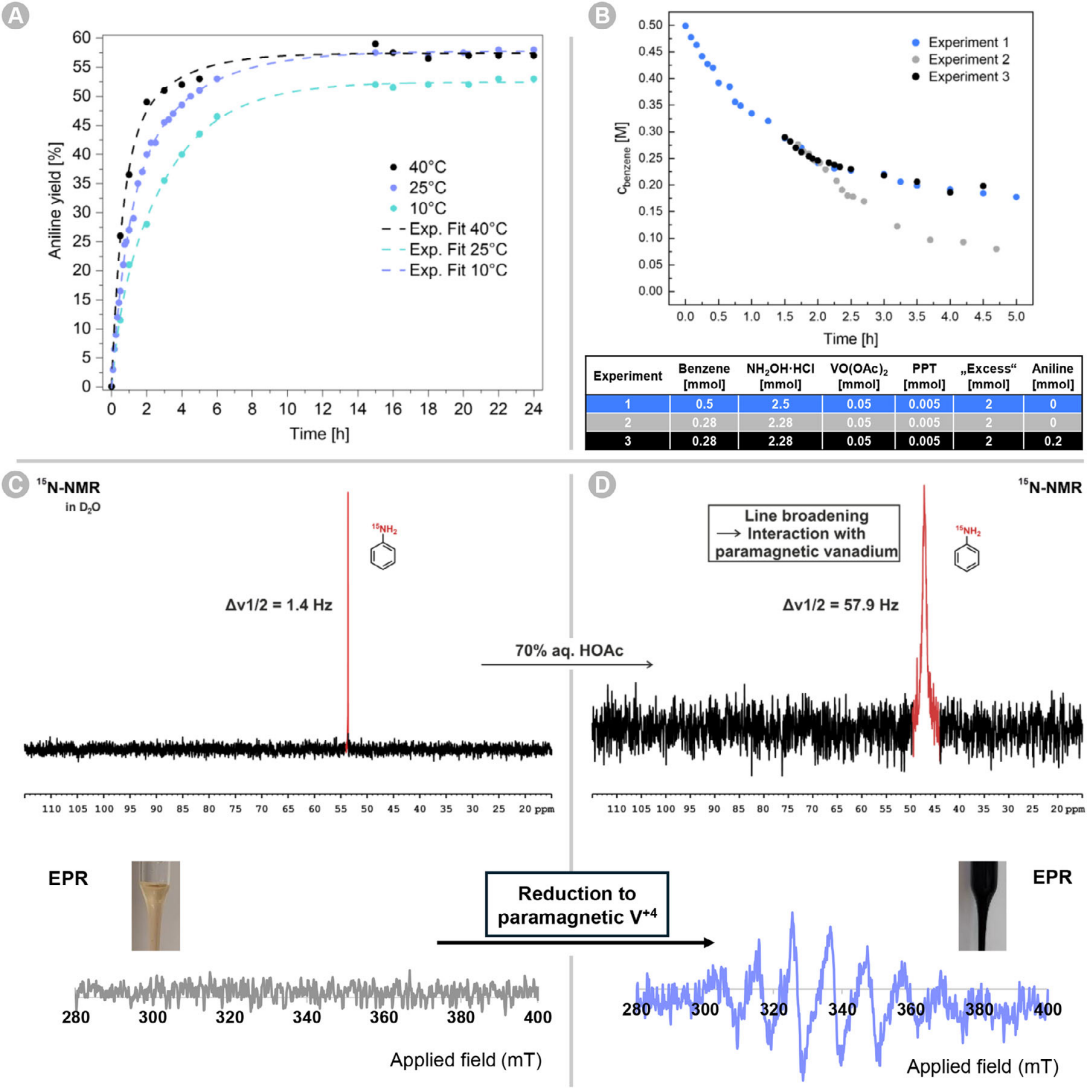
(A) Temperature effect on the kinetic profile of benzene amination. Lower yields obtained at 10°C correspond with lower solubility of the reaction components. For both reaction at higher temperature, the cut‐off for the aniline yield is identical at around 60% with only a slightly slower rate for 25°C. Reaction conditions: 0.5 mmol benzene, 5 equiv. NH_2_OH·HCl, 10 mol% VO(OAc)_2_, 1 mol% PPT, 1 mL aq. AcOH, LED (365 nm). Yields were determined by GC‐FID with toluene as internal standard. (B) Reaction progress of benzene amination using the same excess of hydroxylamine, but different substrate concentrations in 1 mL 70% aq. AcOH. Initial benzene concentration of 0.5 M (violet plot), initial benzene concentration of 0.28 M (grey plot), initial benzene concentration of 0.28 M with 0.2 mmol aniline (black plot). No overlay of the grey and blue curves suggests the catalyst deactivation. After addition of aniline, the overlay confirmed the catalyst inhibition by the product, resulting in a plateau in the kinetics after certain period. (C) NaVO_3_ (0.05 M) + aniline (2 equiv.) in D_2_O (NMR: NS = 2k; EPR: *ν*_mw_ = 9.458819 GHz). With no acetic acid present, the aniline signal shows no broadening in the ^15^N‐NMR and no paramagnetic species were detected in the EPR. D: NaVO_3_ (0.05 M) + aniline (2 equiv.) in 70% aq. AcOD‐d4 after filtration (NMR: NS = 2k; EPR: *ν*_mw_ = 9.460723 GHz). With aq. acetic acid, the aniline signal exhibits major broadening in the ^15^N‐NMR and some additional paramagnetic species in the EPR, indicating interaction of aniline with the vanadium catalyst in the reaction medium.

Since such amination reactions can also proceed under purely thermal conditions [37], we examined whether temperature affects the photocatalytic benzene amination. Reactions were carried out at 10°C, 25°C, and 40°C (Figure 5A). While higher temperatures slightly accelerated the reaction, final yields at 25°C and 40°C were identical. In contrast, the reaction at 10°C proceeded more slowly and gave lower yields, likely due to reduced solubility of the reactants. Despite the clean reaction profile, kinetic experiments revealed a plateau at around 60% yield. To investigate this behaviour, kinetic studies following the methodology of Blackmond were performed [63, 64]. In the “same excess” experiments, two reactions were run with an identical excess of hydroxylamine (2 mmol), but different initial benzene concentrations. After normalising and time‐shifting the curves, no overlay was observed (Figure 5B, blue and grey), indicating either catalyst deactivation or product inhibition. To distinguish between these, 0.2 mmol of aniline was added to the reaction mixture containing 0.28 M benzene, mimicking the composition after 1.5 h in the original experiment with 0.5 M benzene. In this case, the curves overlapped after time correction (Figure 5B, blue and black), demonstrating catalyst robustness and confirming that product inhibition by aniline is responsible for the observed reaction ceasing.

To elucidate the origin of the inhibition, spectroscopic studies were conducted. In aqueous solution of aniline and the vanadium complex, neither significant broadening of the aniline resonance in the ^15^N‐NMR spectrum, nor EPR‐active species were detected (Figure 5C). On the contrary, the aniline resonance broadened significantly (57.9 Hz) in acetic acid, and the paramagnetic species appeared in the EPR spectrum (Figure 5D), arising from the partial reduction of V^+5^ to V^+4^ by aniline. These observations indicate the interaction of aniline with vanadium species in acetic acid and can explain the inhibitory effect detected during the kinetic investigations.

### 2.4 Reaction Mechanism

**FIGURE 6:**
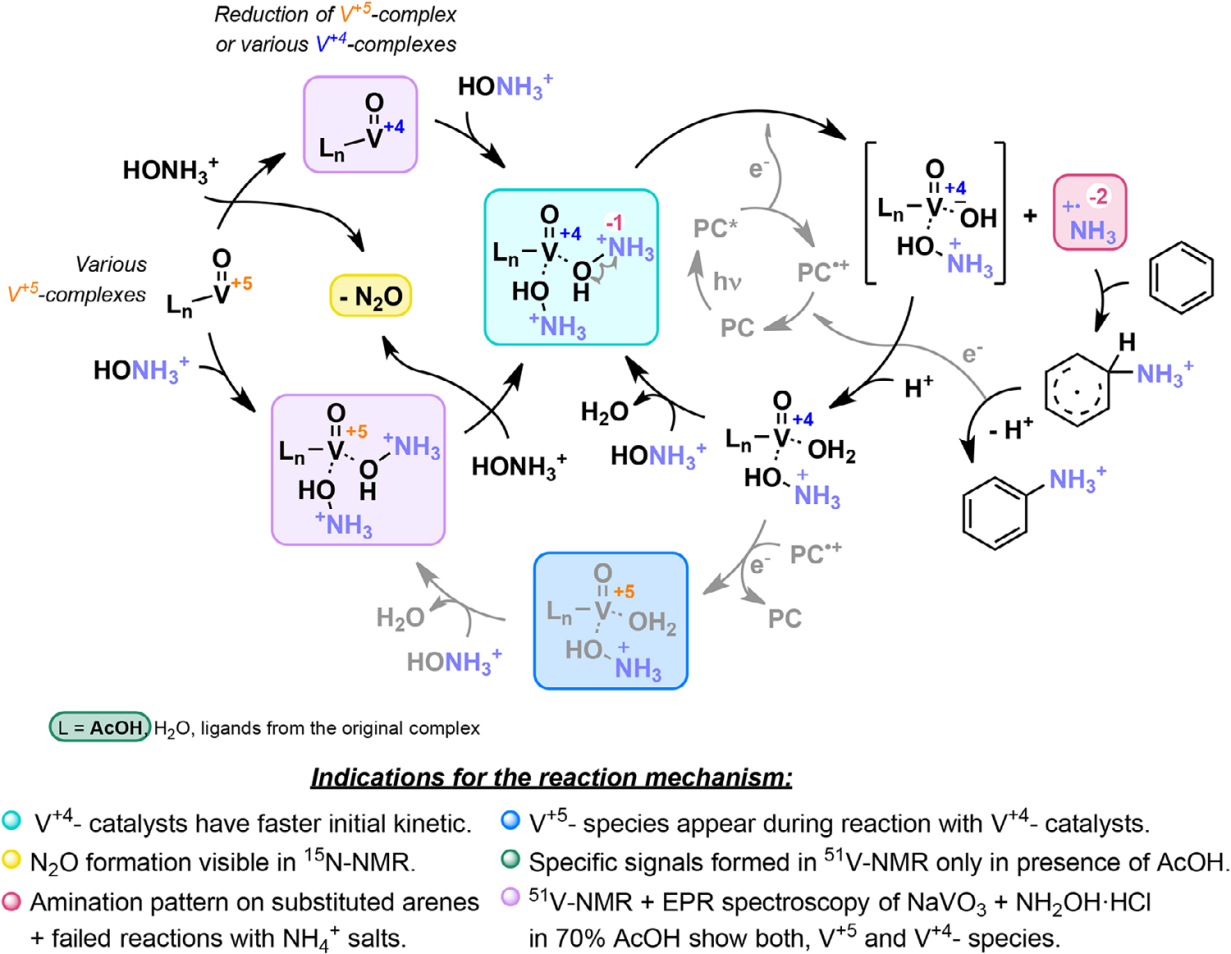
Proposed reaction mechanism of the direct photocatalytic amination of benzene. L = AcOH, H_2_O, ligands from the original complex; *n* = 1–3. The addition of hydroxylamine to vanadium V^+4^ and V^+5^ precursors leads to the formation of the active vanadium(IV)‐hydroxylamine complex either by ligand exchange or reduction (turquoise rectangle). From here, a photocatalytic generated NH_3_^+^∙ radical cation attacks benzene to form aniline. The active intermediate is regenerated by ligand exchange or oxidation/and ligand exchange followed by a reduction.

Based on previous literature report [37], which proposes a free radical mechanism for the thermal approach, and our spectroscopic investigation, we propose the following mechanism (Figure 6). In the initial phase, the vanadium catalyst reacts with hydroxylamine to form the previously described bis‐hydroxylamine‐vanadium complexes (Figure 2A). For V^+5^‐catalysts, this species can arise either by ligand exchange followed by reduction of the V^+5^‐complex, or by initial reduction by hydroxylamine followed by the ligand exchange (Figure 3). For V^+4^ precursors, hydroxylamine can directly coordinate via oxygen to the V^+4^‐centre only through ligand exchange (Figure 6, turquoise rectangle). The detected N_2_O (Figure 6, yellow rectangle) is released during the reduction of the V^+5^‐complex by hydroxylamine and its decomposition (Figure S20), whereas with the V^+4^ catalyst hydroxylamine signal remains intact, indicating direct coordination (Figure S27). Kinetics studies indicate the vanadium(IV)‐hydroxylamine complexes as the catalytically active species by exhibiting a slower initial rate (Figures 4B, C).

Next, the active intermediate undergoes single‐electron reduction at nitrogen, resulting in cleavage of the activated N–O bond. This transformation is facilitated by the photocatalyst. The released NH_3_^+^∙ radical cation (Figure 6, pink square) subsequently reacts with the aromatic substrate and, after oxidation and re‐aromatization, affords the aminated product. The observed regioisomer distribution for substituted arenes (Figure 1C), which lies between an electrophilic aromatic substitution and a radical pathway, is consistent with NH_3_^+^· acting as the reactive intermediate. In addition, the failed reactions with ammonium salts (Figure 1A, **entries 16, 17**) exclude the amination by NH_4_^+^ ion. After N─O bond cleavage, the remaining OH coordinated to vanadium can be protonated by the solvent. The resulting water ligand is then exchanged by hydroxylamine, thereby regenerating the active intermediate (Figure 6, turquoise rectangle). Concurrently, the V^+4^‐water complex is oxidised by PC^·+^ to form a vanadium(V)‐monohydroxylamine complex (Figure 6, blue rectangle), which subsequently undergoes ligand exchange to form the bis‐hydroxylamine complex (Figure 6, violet rectangle). This off‐cycle pathway is supported by ^51^V‐NMR spectra recorded during VO(acac)_2_‐catalyzed benzene amination, which revealed the formation of V^+5^‐mono‐ and V^+5^‐bishydroxylamine complexes (Figure 4D, left). Moreover, ^51^V‐NMR monitoring of NaVO_3_ under irradiation showed that the photocatalyst regenerates the vanadium(V)‐bis‐hydroxylamine species, whereas in its absence these signals gradually diminish (Figure S18).

## 3 Conclusion

This study demonstrates the efficient amination of benzene by hydroxylamine at room temperature under acidic conditions. A dual catalytic system, comprising various vanadium complexes in different oxidation states and *N*‐phenylphenothiazine as a photocatalyst, was utilized. The use of photochemistry enables milder reaction conditions, thereby enhancing the overall efficiency of the process. Despite a limited substrate scope, the protocol exhibited excellent selectivity for the amination of nonprefunctionalized aromatic compounds, affording the corresponding anilines in good yields. Mechanistic ^51^V‐NMR spectroscopy investigations provide evidence for the coordination of two hydroxylamine molecules to the vanadium(V) catalyst under reaction conditions. Additionally, the experimental data indicate a crucial role of acetic acid: it may act as a solvent, proton source, and ligand, thereby modulating the vanadium bis‐hydroxylamine complexes by enabling dynamic coordination exchange and maintaining accessibility for productive catalysis. While NMR spectroscopy suggested the formation of distinct vanadium(V)‐bis‐hydroxylamine complexes, EPR spectroscopy revealed a partial, time‐dependent reduction of V^+5^ to V^+4^ by hydroxylamine. Reaction‐kinetic studies employing ^1^H‐, ^51^V‐, and ^15^N‐NMR further highlighted the critical involvement of vanadium(IV)‐hydroxylamine complexes as active intermediates, as indicated by slower initial reaction rates when using vanadium(V) pre‐catalysts. The photocatalyst facilitates reductive N–O bond cleavage and helps sustain the active redox cycle. Moreover, same‐excess kinetics and ^15^N‐NMR studies revealed catalyst deactivation through aniline inhibition, leading to plateaued yields. Collectively, these mechanistic investigations emphasise the intricate interplay between coordination chemistry, solvent effects, and redox balance in the vanadium‐mediated photocatalytic reactions. The proposed mechanism involves an ammonium radical cation attack on arenes following the photocatalytic N─O bond cleavage after the coordination and activation of hydroxylamine through vanadium. The unique coordination chemistry and redox versatility of vanadium are essential for the successful selective aromatic amination under mild conditions. A deeper understanding of these variables is crucial for further optimisation of the photocatalytic process, paving the way for broader applications.

## Conflicts of Interest

The authors declare no conflicts of interest.

## Supporting information




**Supporting File**: The authors have cited additional references within the Supporting Information [37, 56, 62, 65–69].
