# Supplementary material for: Photocatalytic Vanadium‐Mediated Amination of Benzene With Hydroxylamine
Source: Chemistry. 2026 Mar 28;32(22):e03475. doi: 10.1002/chem.202503475 (PMC13250366; doi:10.1002/chem.202503475)
Supplement: Supplementary file 1 — Supporting File: The authors have cited additional references within the Supporting Information [37, 56, 62, 65–69]. [file CHEM-32-e03475-s001.docx]

**Electronic Supplementary Information**

Photocatalytic Vanadium-mediated Amination of Benzene with Hydroxylamine

Dana Králová^[a]^, Maximilian Philipp^[a]^, Matthias Schmalzbauer^[b]^, Thilo Reiter^[a]^, Bernd Schäfer^[b]^, Ruth M. Gschwind^*[a]^, Burkhard König^*[a]^

[a] Fakultät für Chemie und Pharmazie, Universität Regensburg, 93053, Regensburg, Germany

[b] BASF SE, Carl-Bosch-Straße 38, 67056 Ludwigshafen am Rhein, Germany

The correspondence should be addressed to: [Ruth.Gschwind@chemie.uni-regensburg.de](mailto:Ruth.Gschwind@chemie.uni-regensburg.de) (R.M.G) and [Burkhard.Koenig@chemie.uni-regensburg.de](mailto:Burkhard.Koenig@chemie.uni-regensburg.de) (B.K)

Primary research data are available at: **10.5281/zenodo.17456000**

Table of Contents

[General information 3](#_Toc213068711)

[Experimental procedures 6](#_Toc213068712)

[Optimisations 7](#_Toc213068713)

[UV-VIS spectroscopy 11](#_Toc213068714)

[NMR spectroscopy 12](#_Toc213068715)

[EPR spectroscopy 25](#_Toc213068716)

[Effect of LED radiant power and GC analysis 26](#_Toc213068717)

[References 27](#_Toc213068718)

# General information

All reactions were carried out in degassed solvents (by freeze-pump-thaw method) stored under nitrogen atmosphere unless noted otherwise. Solvents and reagents required were purchased from commercial suppliers and used without further purification.

**UV–Vis** measurements were performed with a Cary 4000 spectrometer.

**Gas chromatography** measurements were performed on a GC 7890 by Agilent Technologies equipped with flame-ionisation detector (FID) and analysed at the Agilent Chem Station Rev.C.01.04. GC coupled to low-resolution mass spectrometry (GC-MS) measurements were performed on 7890A GC-System by Agilent Technologies equipped with Agilent 5975 MSD single quadrupole detector and analysed at the MSD Chem Station E.02.02.1431. The capillary column used was an HP-5MS/30 m x 0.25 mm/0.25 µM film with helium as the carrier gas (flow rate: 1 ml/min). The GC measurements were analysed by integrating the obtained signal. The GC temperature program was set as follows: the temperature of 40 °C was maintained for 3 min. Subsequently, the temperature was increased at a rate of 15 °C/min over a period of 16 min until 280 °C was reached. This temperature was maintained for 5 min before the temperature was raised to 300 °C at a rate of 25 °C/min over a period of 48 sec and maintained at 300 °C for 5 min.

All reaction yields were determined by calibrated GC-FID using toluene as an internal standard.

**NMR spectroscopy**

**General preparation of oxygen-free NMR samples with and without *in situ* illumination**

For sample preparation under protection gas, a Schlenk technique was employed, and argon was used as the protective gas (**Figure S1**).


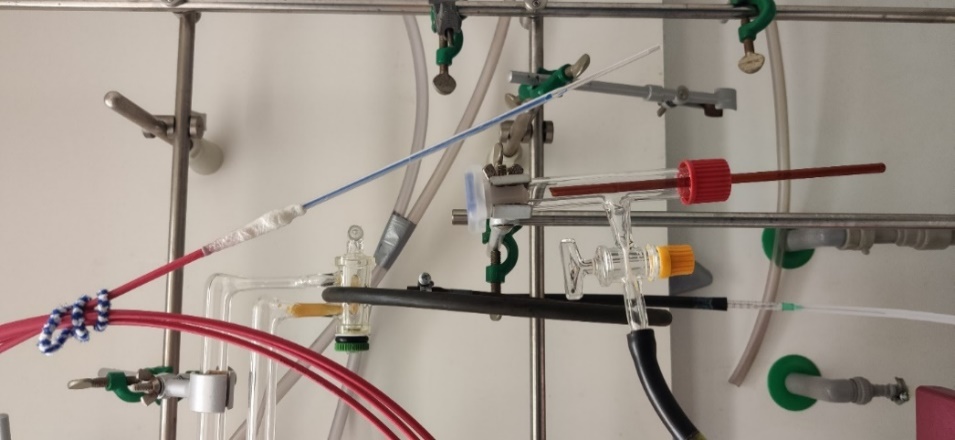


***Figure S1****: Preparation of the NMR sample under an inert gas atmosphere. The NMR tube undergoes three cycles of reduced pressure after the addition of the starting materials. Subsequently, the glass fibre/NMR cap is inserted using argon countercurrent to ensure an oxygen-free sample.*

Initially, solid starting materials were weighed in a dry NMR tube. Then, the liquid reagents were added under a protective gas using the appropriate Gilson direct displacement syringes MICROMAN E M100E, 10-100 µL, MICROMAN E M100E, 1-10 µL, MICROMAN E M100E, 100-1000 µL. After adding the individual reaction components, the pressure was reduced to 0.1 mbar and then re-flooded with argon three times to ensure a protective gas atmosphere. For *in* *situ* illumination measurements under argon, a glass fibre with a suitable insert for the NMR tube was placed in the NMR tube filled with the reaction solution and secured with Parafilm. (**Figure S1**) For standard NMR measurements, a cap with addition of Parafilm was placed on the tube with argon atmosphere.

**General setup for the *in situ* illumination inside the spectrometer**

For the illumination within the NMR spectrometer, the *in situ* illumination setup developed by the Gschwind group *et al.* was employed.^[1]^ The glass fibre, housing an insert within the NMR tube, is linked to an LED within a custom-built illumination chamber.

For illumination of the NMR samples, the LED New Energy LST1-01G01-UV01-00 was utilised as a light source with an effective peak wavelength of 365 nm, operating at the current of 600 mA.

All NMR spectroscopic investigations on model systems were performed on a Bruker Avance III HD 600 MHz spectrometer with TBI (Triple resonance broadband inverse) 5 mm CPPBBO 1H/19F-BB probe head with Z-gradient and BVT unit, and with PI HR-TBO (Triple resonance broadband observe) 600S3-BBER/H/N/D-5.0-Z DPT probe head. Temperature was controlled by a BVT 3900 unit and liquid nitrogen.

Processing of the NMR spectra was executed in Topspin 3.2. The spectra were phased, and the baseline was corrected. Data were analysed in Microsoft Excel and Origin 2020. Figures were prepared in the Origin 2021.

^1^H chemical shifts were if stated referenced to TMS or the respective solvent residual signals. The ^51^V-heteronucleus could be referenced, employing ν(X) = ν(TMS) · Ξreference / 100 % according to Harris et al.^[2]^ The following frequency ratio and reference compounds were used: Ξ(^51^V) = 26.302948 (VOCl_3_). Due to the similarity of vanadium NMR shifts and no change in qualitative interpretation with and without any referencing no external referencing is the preferred method. Also, the known peaks are set in accordance with the literature values.^[3]^

**EPR spectroscopy**

EPR spectra of vanadium radical species were measured on a Magnettech Miniscope MS 400 spectrometer (9.45 GHz, X-band) at 20 °C. The samples were prepared under inert conditions in flame-sealed Pasteur pipettes.

**Photoreactor**

Reactions were irradiated with 365 nm LEDs (SSC VIOSYS CUN66A1B, average radiant flux 0.55 ± 0.05 W, 3.6 V, 0.7 A; or IN‑C68QABTMU2 UV, average radiant flux 2.97 ± 0.10 W, 14 V, 0.7 A). An aluminium block connected to the thermostat was used for temperature control during the reaction time (**Figure S2**). The radiant flux of LEDs (**Table S1**) was measured with FieldMaxII-TOTM Laser Power Meter with PM3 Sensor.

**Figure S2:** Setup used for photochemical reactions in batch. A: Thermostat connected to the aluminium block. B: Aluminium block and the LED plate on the magnetic stirrer. C: LED plate.

**Table S1:** Overview of radiant fluxes of LEDs used for the reactions.

| **LEDs** | **Radiant flux stated by manufacturer @700 mA** | **Measured radiant flux** |
| --- | --- | --- |
| Standard power LEDs  SSC VIOSYS CUN66A1B | 1250 mW | 550 ± 50 mW |
| High power LEDs IN‑C68QABTMU2 | 3000 mW | 2973 ± 100 mW |

The emission spectrum of the LEDs (**Figure S3**) was measured with Ocean Optics HR4000CG‑UV-NIR glass fibre and diffuser.

**Figure S3:** Emission spectrum of the used LEDs.

**Microflow reactor**

The microflow setup (**Figure S4**) consisted of the syringe pump LA-30 from Landgraf HLL connected by Teflon tubing to the microreactor MR-LAB-V by Little Things Factory GmbH (size: 115 x 60 x 6 ± 0.5 mm (l, w, h), internal volume 1.7 mL, channel size: 1 mm, transmission of light ~ 91%) placed in a cooling block. Irradiation was enabled from the top by six LEDs (SSC VIOSYS CUN66A1B, average radiant flux 0.55 ± 0.05 W, 3.6 V, 0.7 A).


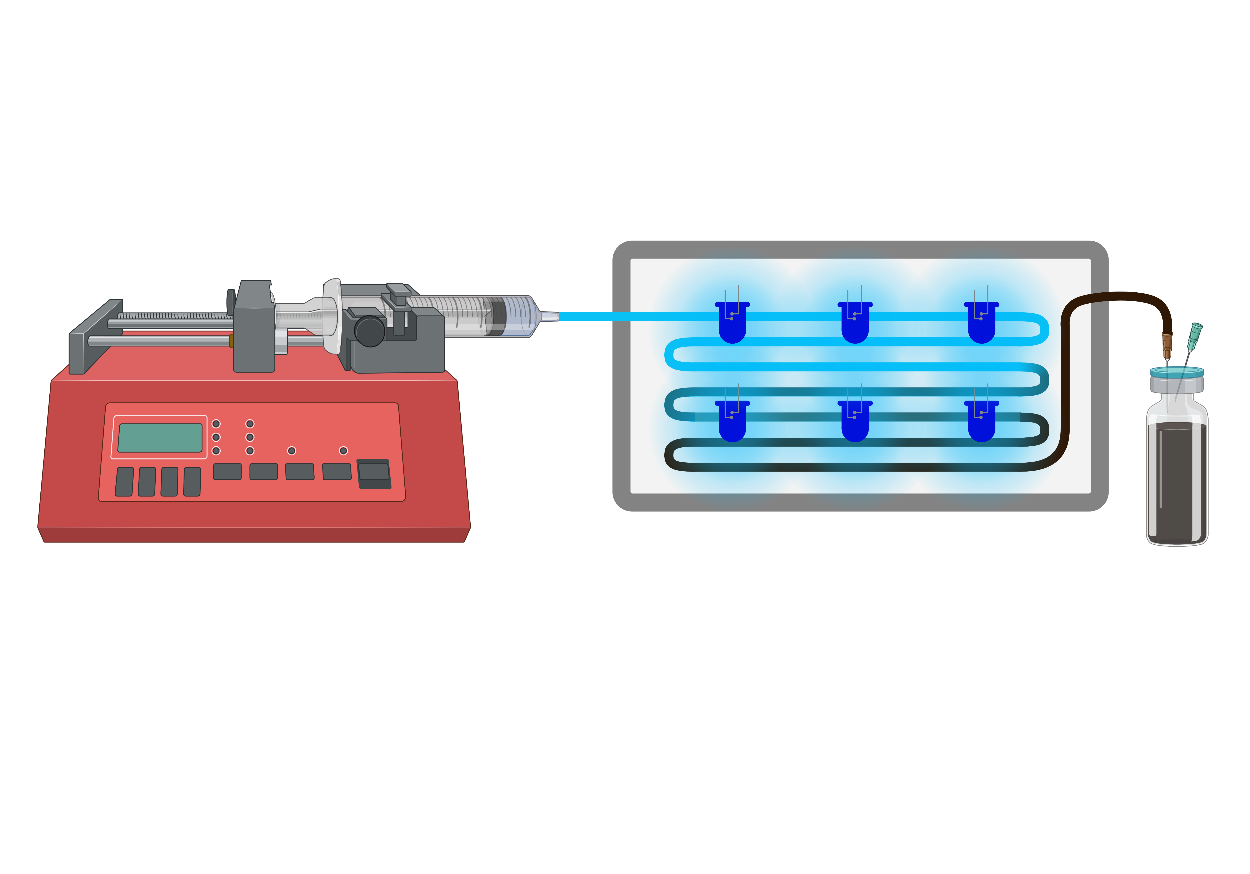


**Figure S4:** Microflow setup used for the amination of benzene. (Created with [BioRender.com](https://www.biorender.com/))

# Experimental procedures

**Amination reaction in batch**

*N*-Phenylphenothiazine (1.4 mg, 0.005 mmol, 0.01 equiv.), vanadium catalyst (0.05 mmol, 0.1 equiv.), hydroxylamine hydrochloride (174 mg, 2.5 mmol, 5 equiv.), and stirring bar were added into a 5 mL crimp vial. The vial was capped, and the atmosphere was exchanged to N_2_ by the standard Schlenk technique. Degassed solvent mixture (1 mL) was added under nitrogen with a syringe, followed by benzene (45 μL, 0.5 mmol, 1 equiv., stored under nitrogen). The resulting heterogenous mixture was stirred until most of the components dissolved (⁓ 10 min). Subsequently, the vial was placed into a metal block connected to a thermostat, and the solution was irradiated with an LED (wavelength and radiant power noted in **Table S1** and **Figure S3**) for a certain time. After the reaction time, the mixture was transferred to a 15 mL centrifuge test tube and adjusted to neutral/slightly basic pH by the addition of 3 M aq. NaOH. The resulting aqueous mixture was extracted with diethyl ether, and subsequently, toluene (40 μL, 0.38 mmol) was added as an internal standard for the subsequent GC-FID analysis.

**Amination of benzene in microflow reactor**

*N*-Phenylphenothiazine (4.2 mg, 0.015 mmol, 0.01 equiv.), vanadyl acetoacetonate (41.0 mg, 0.15 mmol, 0.1 equiv.), hydroxylamine hydrochloride (521 mg, 7.5 mmol, 5 equiv.), and stirring bar were added to the 10 mL crimp vial. The vial was capped, and the atmosphere exchanged to N_2_ by the standard Schlenk technique. Degassed water (0.9 mL), glacial acetic acid (2.1 mL), and benzene (135 μL, 1.5 mmol, 1 equiv., stored under nitrogen) were added to the vial under nitrogen. The heterogenous mixture was stirred at room temperature until completely homogeneous light blue solution was formed (1-2 h).

The micro flow reactor was flushed with nitrogen, and the reaction mixture was transferred into the gas tight syringe (Omnifix^®^ 5mL, Luer Lock Solo, inner diameter = 12.5 mm), which was fixed in the syringe pump, and the flow rate was set to 1.72 mL/h. The reaction mixture was irradiated inside the flow reactor with five 365 nm LEDs (~ 500 mW), and the solution was collected into an empty vial.

Aliquot of 1 mL from the collecting vial was transferred to a 15 mL centrifuge test tube followed by the same workup and analysis as for the batch reactions.

# Optimisations

***Table S2:*** *Optimisation of reaction conditions and control experiments for amination of benzene in the presence of vanadium catalysts.*

| **Entry** | **Vanadium-catalyst** | **Vanadium-catalyst (mol%)** | **NH_2_OH·HCl (equiv.)** | **Aniline yield (%)** | **Notes** |
| --- | --- | --- | --- | --- | --- |
| 1 | NaVO_3_ | 0 | 5 | 0 | - |
| 2 | NaVO_3_ | 2 | 5 | 36 | - |
| 3 | NaVO_3_ | 5 | 5 | 45 | - |
| 4 | NaVO_3_ | 10 | 5 | 49 | - |
| 5 | NaVO_3_ | 15 | 5 | 50 | - |
| 6 | NaVO_3_ | 10 | 1 | 19 | - |
| 7 | NaVO_3_ | 10 | 3 | 38 | - |
| 8 | NaVO_3_ | 10 | 7 | 49 | - |
| 9 | NaVO_3_ | 10 | 5 | 0 | air |
| 10 | NaVO_3_ | 10 | 5 | 0 | no light |
| 11 | NaVO_3_ | 10 | 5 | 9 | no PPT |
| 12 | NaVO_3_ | 10 | 5 | 0 | no PPT, no light |
| 13 | NaVO_3_ | 10 | 5 | 5 | no PPT, 25 °C |
| 14 | V(acac)_3_ | 10 | 5 | <1 | no PPT, 25 °C |
| 15 | VOSO_4_·5 H_2_O | 10 | 5 | 9 | no PPT, 25 °C |
| 16 | VO(OAc)_2_ | 10 | 5 | 20 | no PPT, 25 °C |
| 17 | VO(acac)_2_ | 10 | 5 | 5 | no PPT, 25 °C |
| 18 | VO(OEt)_3_ | 10 | 5 | <1 | no PPT, 25 °C |
| 19 | V_2_O_5_ | 10 | 5 | 0 | no PPT, 25 °C |
| 20 | VO(PcPhO) | 10 | 5 | 0 | no PPT, 25 °C |

Screening of the photocatalysts (**Table S3**) indicate that the catalytic performance does not correlate simply with excited-state reduction potentials, but rather with the ability of the photocatalyst to sustain the operative vanadium redox cycle under the strongly acidic and coordinating reaction conditions. While the simple phenothiazine can disturb the availability of the vanadium catalyst, Miyake catalyst suffers from low solubility in the aqueous medium due to high aromaticity. In addition, the stability of catalysts in acidic medium can also play a role.

**Table S3:** Optimisation of reaction conditions using various photocatalysts, solvent mixtures, and metal catalysts. Photocatalysts and metal catalysts were optimised in 70% aq. AcOH. ^*^ Irradiation wavelength noted under the photocatalysts. ^a^ 5 mol% VO(acac)_2_ as metal catalyst. ^b^ 10 mol% NaVO_3_ as metal catalyst. ^c^ 1 mol% PPT as photocatalyst. ^d^ Reaction was performed at 25 °C instead of 40 °C.

**Table S4:** Amination of benzene using different acids at c_acid_=12.8 mol/L. Reaction conditions: 0.5 mmol benzene, 5 equiv. NH_2_OH·HCl, 10 mol% NaVO_3_, 1 mol% N-phenylphenothiazine, 1 mL aq. acid, 40 °C, 4 h, LED (365 nm).

| **Entry** | **Acid** | **Calculated pH of solvent mixture** | **Aniline yield (%)** | **Colour of the reaction mixture before irradiation** |
| --- | --- | --- | --- | --- |
| **1** | AcOH | 1.84 | 49 | 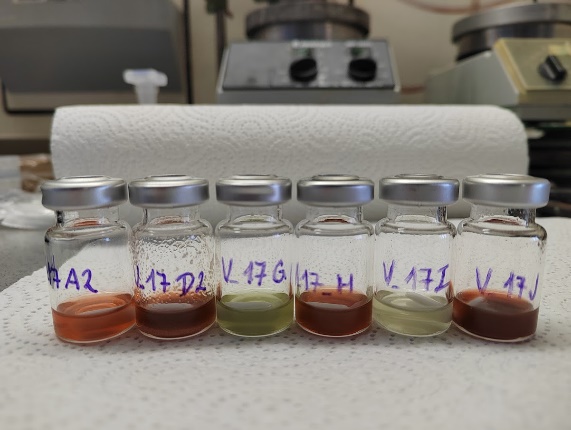 |
| **2**^*^ | (COOH)_2_ | 0.26 | 3 | 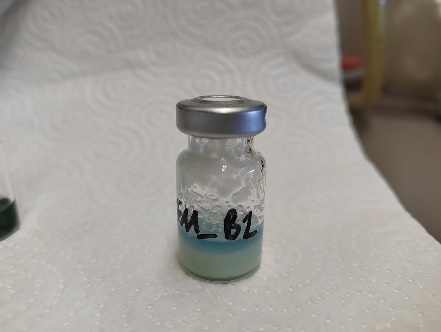 |
| **3** | MeSO_3_H | -1.09 | 2 | 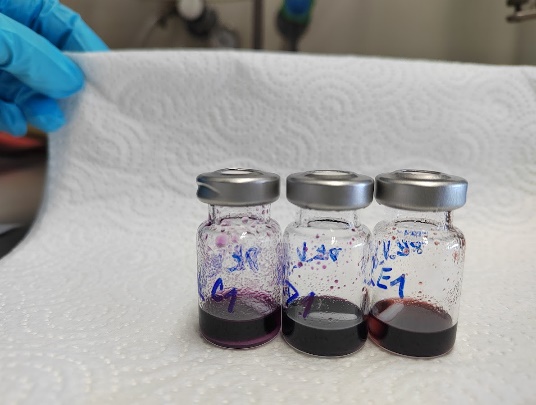 |
| **4** | HCl | -1.09 | 2 | 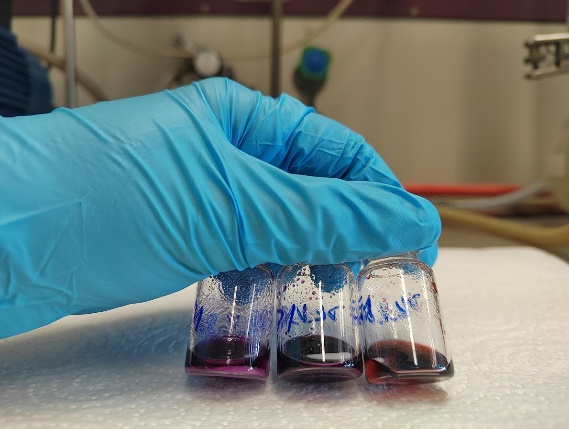 |
| **5** | H_2_SO_4_ | -1.39 | 1 | 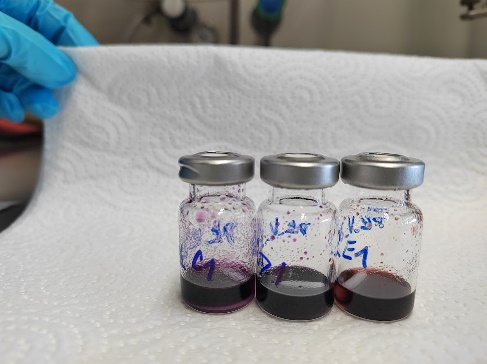 |
| **6** | H_3_PO_4_ | 0.54 | 2 | 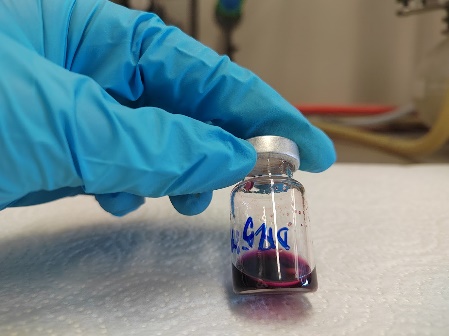 |
| **7^*^** | PivOH | 2.81 | 0 | 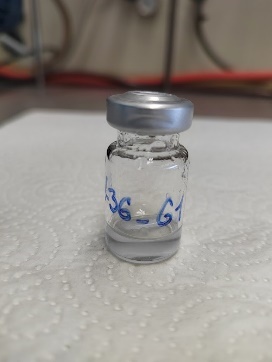 |
| **8** | CF_3_COOH | -1.09 | 0 | 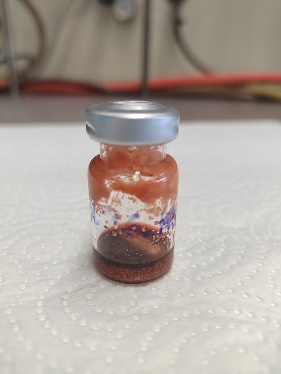 |

^*^Due to the low solubility of oxalic and pivalic acid in water, only 6.1 mol/L and 0.24 mol/L, respectively, was used. In the case of oxalic acid, a certain amount of acid remained undissolved.

**Table S5:** Amination of benzene using different acids at pH = 1.84. Reaction conditions: 0.5 mmol benzene, 5 equiv. NH_2_OH·HCl, 10 mol% NaVO_3_, 1 mol% N-phenylphenothiazine, 1 mL aq. acid, 40 °C, 4 h, LED (365 nm).

| **Entry** | **Acid** | **c_acid_** | **Aniline yield (%)** |
| --- | --- | --- | --- |
| 1 | AcOH | 12.18 M | 49 |
| 2 | HCOOH | 1.19 M | 6 |
| 3 | CH_3_CH_2_COOH | 13.27 M | 8 |
| 4 | ClCH_2_(CH_2_)_2_COOH | 7.09 M | 2 |
| 5 | (COOH)_2_ | 18.3 mM | 3 |
| 6 | CF_3_COOH | 14.8 mM | <1 |
| 7 | MeSO_3_H | 14.5 mM | 1 |
| 8 | HCl | 14.5 mM | 1 |
| 9 | H_2_SO_4_ | 7.2 mM | 1 |
| 10 | H_3_PO_4_ | 44.7 mM | 2 |

**Table S6:** Amination of benzene using other nitrogen-sources. Reaction conditions: 0.5 mmol benzene, 5 equiv. N-source, 10 mol% NaVO_3_, 1 mol% N-phenylphenothiazine, 1 mL 70% aq. AcOH, 40 °C, 4 h, LED (365 nm).

| **Entry** | **Nitrogen source** | **Aniline yield (%)** |
| --- | --- | --- |
| 0 | NH_2_OH·HCl | 49 |
| 1 | N_2_H_4_·AcOH | <1 |
| 2 | N_2_H_4_·HCl | <1 |
| 3 | 50 wt% NH_2_OH in H_2_O | <1 |
| 4 | 50 wt% NH_2_OH in H_2_O + HCl 1:1 (*n*/*n*) | 47 |
| 5 | (NH_3_OH)_2_SO_4_^*^ | 49 |
| 6 | NH_4_Cl | <1 |
| 7 | NH_4_OAc | 0 |
| 8 | NH_4_OAc + HCl 1:1 (*n*/*n*) | 0 |
| 9 | MeNHOH·HCl | 8 |
| 10 | Et_2_NOH·HCl | 0 |
| 11 | NH_2_OMe·HCl | 0 |
| 12 | MeNHOMe·HCl | 0 |
| 13 |  | 0 |
| 14 |  | 0 |
| 15 |  | 0 |

* 2.5 equiv. were used due to the presence of two hydroxylamine units in one molecule of the sulfate salt.

**Table S7:** Hydroxylation of benzene with hydrogen peroxide. Reaction conditions: 0.5 mmol benzene, 5 equiv. 50% (w/w) H_2_O_2_ in H_2_O, 10 mol% vanadium catalyst, 1 mol% PPT, 0.8 mL of solvent, 25 °C, 4 h.

| **Entry** | **V-catalyst** | **Solvent** | **Phenol yield (%)** |
| --- | --- | --- | --- |
| 1 | NaVO_3_ | AcOH-H_2_O 7:3 | 0 |
| 2 | NaVO_3_ | MeOH | 0 |
| 3 | NaVO_3_ | EtOAc | 0 |
| 4 | NaVO_3_ | H_2_O | 0 |
| 5 | VO(acac)_2_ | MeOH | 0 |
| 6 | VO(acac)_2_ | EtOAc | 0 |
| 7 | VO(acac)_2_ | ACN | 0 |
| 8 | VO(acac)_2_ | H_2_O | 0 |
| 9 | VOSO_4_·5 H_2_O | MeOH | 0 |
| 10 | VOSO_4_·5 H_2_O | H_2_O | 0 |

# UV-VIS spectroscopy

UV-VIS spectra were measured for V(acac)_3_, VO(acac)_2_, NaVO_3_, and their corresponding reaction mixtures (**Figure S5**). However, the problem with different absorption intensities of each oxidation state prevented the determination of all the oxidation states in the reaction mixture.^[4]^ The absorption at 760 nm is unique for VO^2+^, which is in agreement with the EPR spectra. V^V^ absorbs only in the UV region, where other compounds are absorbing as well, therefore, from this measurement, it is not possible to determine if this oxidation state is also present in our reaction mixture.

***Figure S5:*** *Absorption spectrum of each vanadium oxidation state in 70% aq. AcOH and the corresponding reaction mixtures with photocatalyst and benzene.*

# NMR spectroscopy

For the vanadium hydroxylamine complexes alternative diamagnetic formulations (e.g., spin-coupled vanadium(IV)) cannot be entirely excluded. Nevertheless, the exceptionally large chemical shift changes (up to ~300 ppm) indicate pronounced electronic perturbation at the vanadium centre and are most plausibly attributed to ligand coordination. The oxidative sensitivity of the system disfavours a vanadium (III) formulation and renders a predominantly vanadium (V) speciation chemically plausible.^[5]^


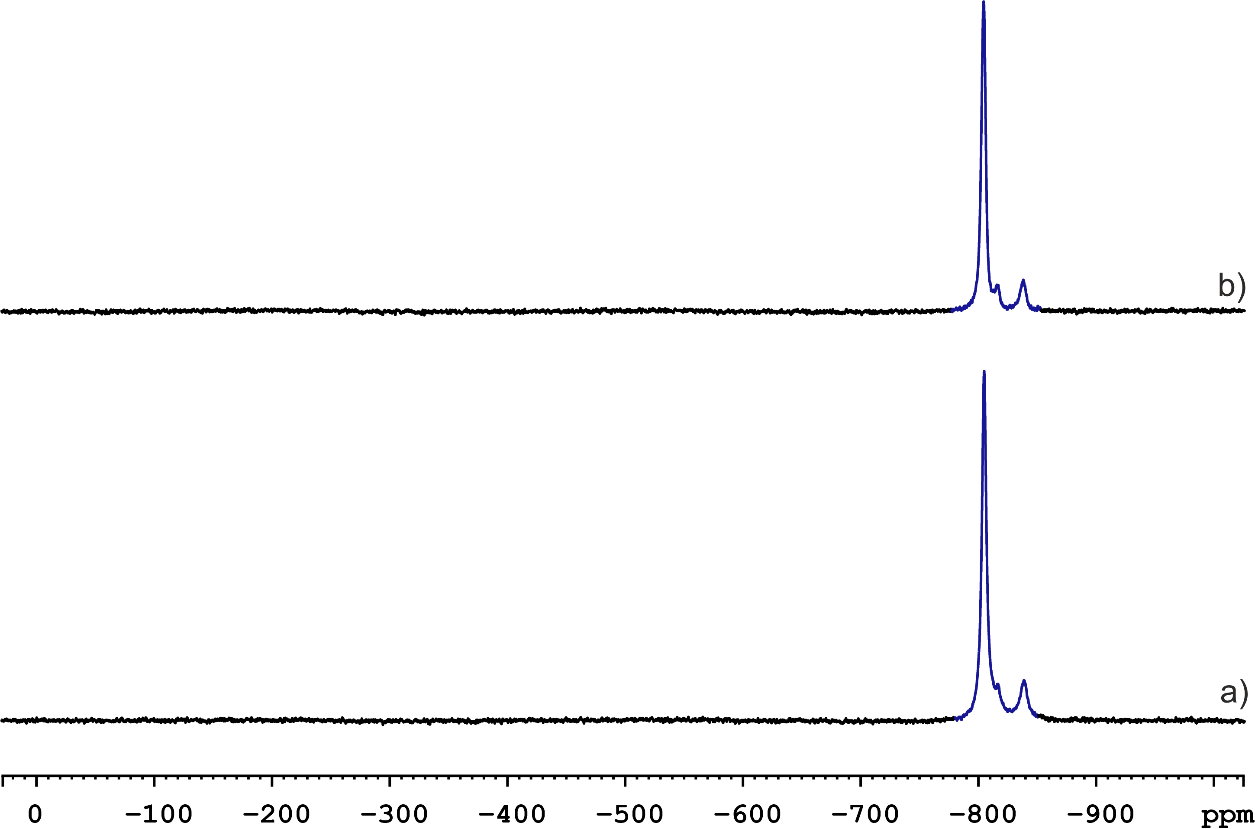


**Figure S6:** ^51^V-NMR spectrum of NaVO_3_ (0.025 M) with 10 equivalents ^15^NH_2_OH∙HCl in DOAc-d_4_/D_2_O 3.5:6.5 with an addition of 600 mg KOAc (1 equiv. regarding to the acetic acid) to create a DOAc/KOAc buffer system with an elevated pH to the very low pH of the conditions (pH≈6). a) no variation of the noted conditions. b) addition of 0.1 mL NaOH to lower the pH to basic conditions (pH≈8) leads to a similar spectrum. 600 MHz, 298K, pulsprog: zg, NS: 512.

Control ^15^N-NMR spectra of hydroxylamine and acetic acid recorded in the absence of vanadium showed no detectable side reactions or intermediates, such as ester formation (**Figure S7**).


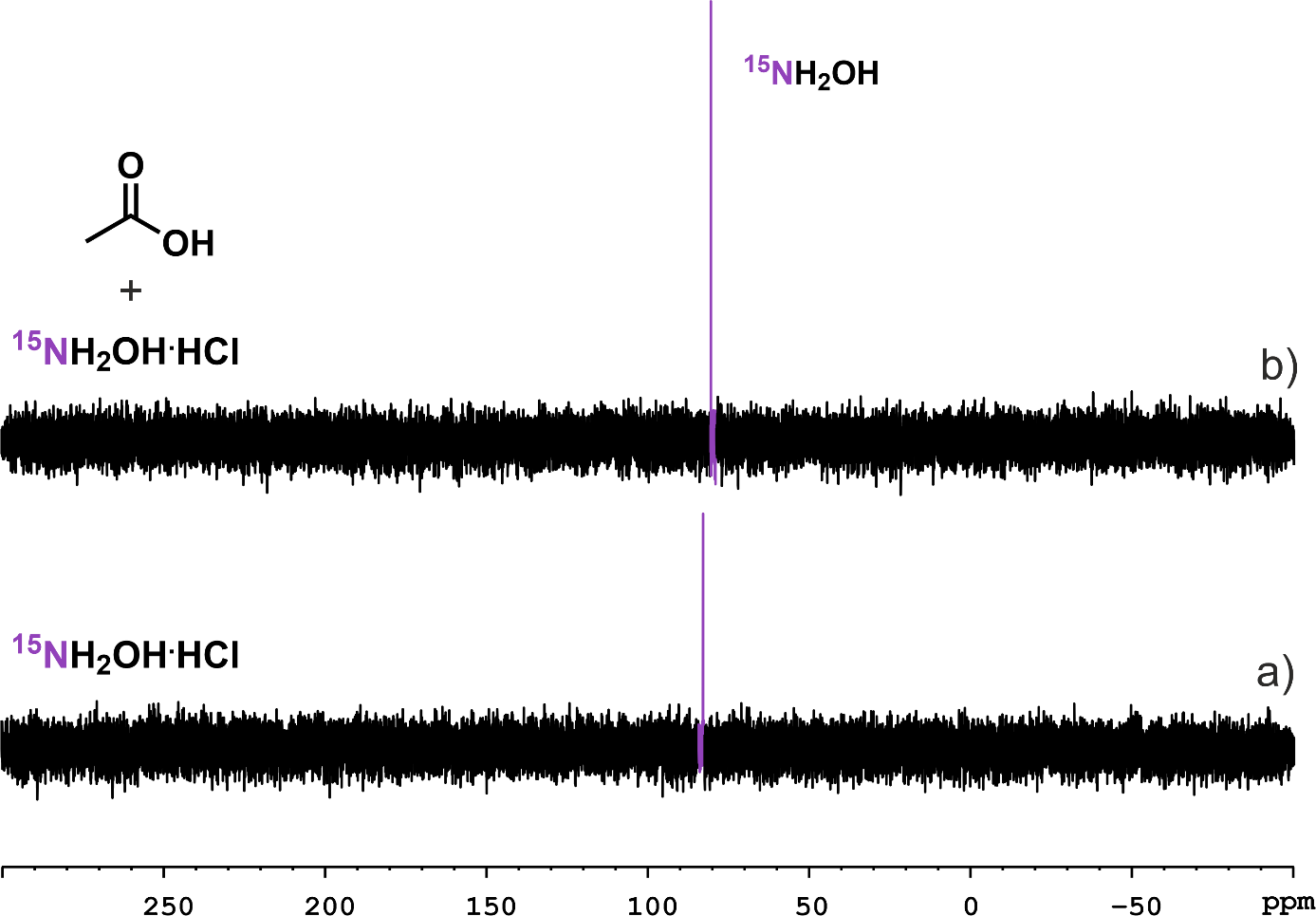


**Figure S7:** ^15^N-NMR reference spectrum of ^15^NH_2_OH∙HCl in D_2_O. a) no variation of the noted conditions. b) addition of 0.1 mL (excess) acetic acid (HOAc). HOAc is used as a solvent in the amination reaction. This addition of HOAc leads to a high field shift of approximately 2.5 ppm. 600 MHz, 298K, pulsprog: zgpg30 (^1^H-decoupled), NS: 512.


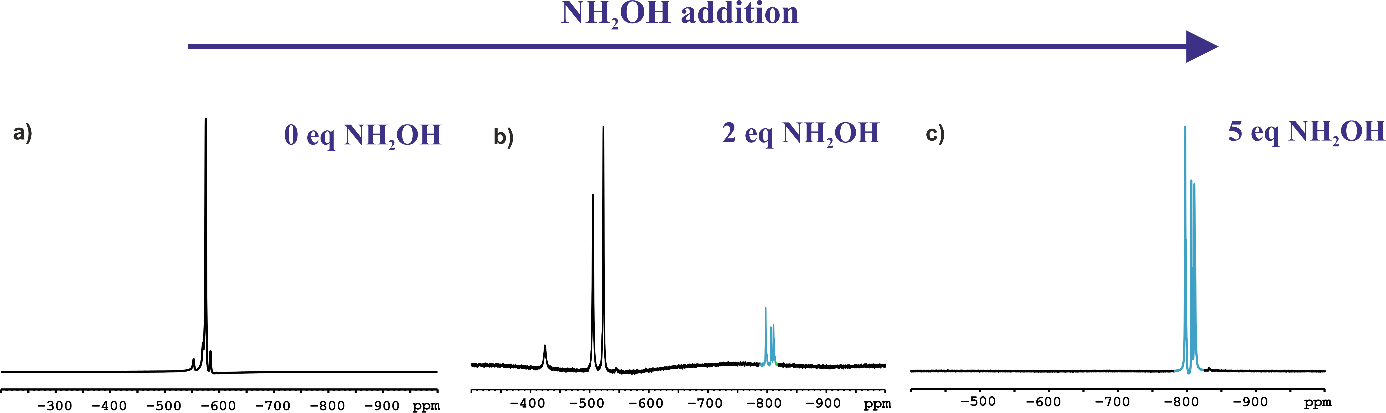


**Figure S8:** ^51^V-Spectra of NaVO_3_ (0.05 M) with varying amounts of NH_2_OH∙HCl in D_2_O/H_2_O (1:9). a) In water free NaVO_3_ appears at around -550 ppm. no NH_2_OH∙HCl. b) Addition of 2 equiv. NH_2_OH∙HCl forms to a minor amount vanadium bis-hydroxylamine complexes, way less compared to the mixture in acetic acid with additional unassigned complexes at around -500 ppm. c) With 5 equiv. NH_2_OH∙HCl in water all vanadium species lie in different vanadium complexes with each 2 molecules of hydroxylamine bound. 600 MHz, 298K, pulsprog: zg, NS: 16k.


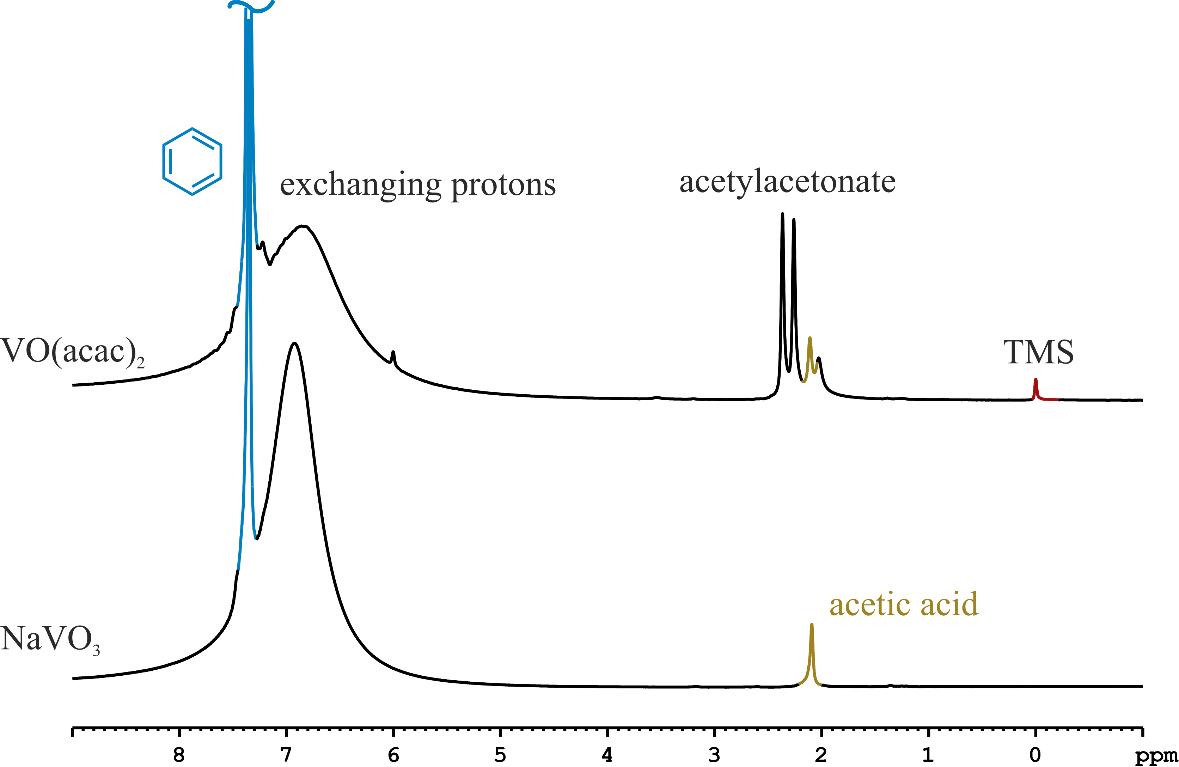


**Figure S9:** ^1^H-NMR spectra of the amination with benzene (0.5 M) with VO(acac)_2_ (upper spectrum, 10 mol%) and NaVO_3_ (lower spectrum, 10mol%), PC (2 mol%) as catalysts and 5 equiv. NH_2_OH∙HCl in deuterated acetic acid/water (7:3). The spectrum with VO(acac)_2_ shows free acetylacetonate which supports ligand exchange. ^1^H-NMR: 600 MHz, 298K, pulsprog: zg, NS: 2.

**^1^H-NMR *in situ* kinetics**

For *in situ* illumination kinetics observed via NMR spectroscopy for proton the PROJECT pulse sequence is used (D2 (cycle time) = 0.001 s, L31 (loop counter) = 32) to eliminate of the broad hydroxylamine peak which overlaps with the aromatic protons of the SM and product. Therefore, the kinetics are not quantitative but qualitative comparable in relation to each other. The TMS peak was referenced to the same integral value for every spectrum. The vanadium catalysts VO(acac)_2_ and NaVO_3_ are selected to compare the influence of a different starting oxidation state.

**Figure S10:** Qualitative kinetic curves derived from ^1^H-NMR of the photocatalytic amination of benzene with the vanadium catalyst VO(acac)_2_ with the oxidation state of V^+IV^ and with the vanadium catalyst NaVO_3_ with the oxidation state of V^+V^. Reaction conditions: benzene (0.5 M), NaVO_3_ (10 mol%), PC (2 mol%) and 5 equiv. NH_2_OH∙HCl in DOAc-d_4_/D_2_O, in situ illumination with LED (365 nm).

Observing both photocatalytic reactions, the reaction rate plateaued at approximately 5-10 hours, consistent with synthetic results. The slower NMR reaction, due to the absence of stirring and decreased light intensities, allowed for more detailed investigations and clearer distinctions between catalysts. Here, the same trends as for the *ex situ* kinetics and experimental yield are observed, where an induction period is present for NaVO_3_. Besides, the overall aniline yield for NaVO_3_ as a vanadium catalyst is higher. The severely increased reaction times and decreased yield most likely arise due to stirring effects and decreased light intensities when using the LED illumination setup^[6]^.

***Ex situ* Illumination Kinetics ^51^V-NMR and ^1^H-NMR**

For *ex situ* illumination kinetics observed via NMR spectroscopy for proton also the PROJECT pulse sequence is used (D2 (cycle time) = 0.001 s, L31 (loop counter) = 32) to eliminate of the broad hydroxylamine peak which overlaps with the aromatic protons of the SM and product. Therefore, the kinetics are not quantitative but qualitative comparable in relation to each other. The NMR parameters are set to the same vales for every measurement to ensure qualitative comparison. Three different vanadium catalyst namely VO(acac)_2_, NaVO_3_ and VOSO_4_ with the oxidation states +4 and +5 were exploited. The benzene peak and aniline peak are normalized to a value of one (assumption is that only minor amounts of benzene are evaporating, similar for each sample and therefore neglectable) for each data point and for the respective corrected rations according to the aromatic protons the following equation is applied:

${correctedIntegral}_{aniline}=\frac{{norm. Intergral}_{aniline} \times6}{norm. {Intergral}_{aniline} \times6 + norm. {Intergral}_{benzene} \times5}$ (1)

${correctedIntegral}_{benzene}=\frac{{norm. Intergral}_{benzene}\times5}{norm. {Intergral}_{aniline} \times6 +norm. {Intergral}_{benzene} \times5}$ (2)

**Figure S11:** Zoomed version of the qualitative kinetic curves derived from ex situ ^1^H-NMR of the photocatalytic amination of benzene with VO(acac)_2_, VOSO_4_, and NaVO_3_. Reaction conditions: 0.5 mmol benzene, 5 equiv. NH_2_OH·HCl, 10 mol% V-catalyst, 1 mol% N-phenylphenothiazine, 1 mL aq. AcOH, LED (365 nm).

**Figure S12:** Qualitative kinetic curves derived from ^1^H-NMR of the ex situ photocatalytic amination of benzene with the vanadium catalysts with the vanadium catalyst NaVO_3_ with the oxidation state of V^+V^. Prereduction of the catalysts for 30 min stirring at room temperature leads to increased conversion within the first hour of the reaction. Reaction conditions: 0.5 mmol benzene, 5 equiv. NH_2_OH·HCl, 10 mol% V-catalyst, 1 mol% N-phenylphenothiazine, 1 mL aq. AcOH, LED (365 nm).

To probe if even further pre-reduction of the vanadium catalyst exhibit faster initial rates here the solution of the different vanadium catalyst are prepared like: The reaction solvent mixture with degassed water:glacial acetic acid (3:7) are put for a minimum of 6 h in the ultrasonic bath at 65 °C till a significant colour change appears and no vanadium(V)-(NH_2_OH)_2_ catalyst is visible in the ^51^V-NMR. Highly reduced conversion and way lower initial rates hint at the formation of unproductive vanadium(IV) species.

**Figure S13:** Qualitative kinetic curves derived from ^1^H-NMR of the ex situ photocatalytic amination of benzene with the vanadium catalysts VOSO_4_ and VO(acac)_2_ with the oxidation state of V^+IV^ and with the vanadium catalyst NaVO_3_ with the oxidation state of V^+V^. Prereduction of the catalysts for a minimum of 6 h at the ultrasonic bath at 65 °C till a significant colour change appears leads to way decreased conversion. Reaction conditions: 0.5 mmol benzene, 5 equiv. NH_2_OH·HCl, 10 mol% V-catalyst, 1 mol% N-phenylphenothiazine, 1 mL aq. AcOH, LED (365 nm).

**^51^V-NMR kinetics**

The vanadium-containing intermediates detected after 30 min reaction time differ primarily in their relative abundances rather than in their qualitative speciation. The overall signal intensity is higher when diamagnetic NaVO₃ is used as the precursor. Notably, for all vanadium precursors investigated, the previously assigned bis(hydroxylamine)-vanadium complexes interacting with acetic acid are observed in the reaction mixture. These observations support a similar, or potentially identical, catalytic cycle operating independently of the vanadium precursor employed, and further emphasise the central role of these intermediate species under the reaction conditions.

**
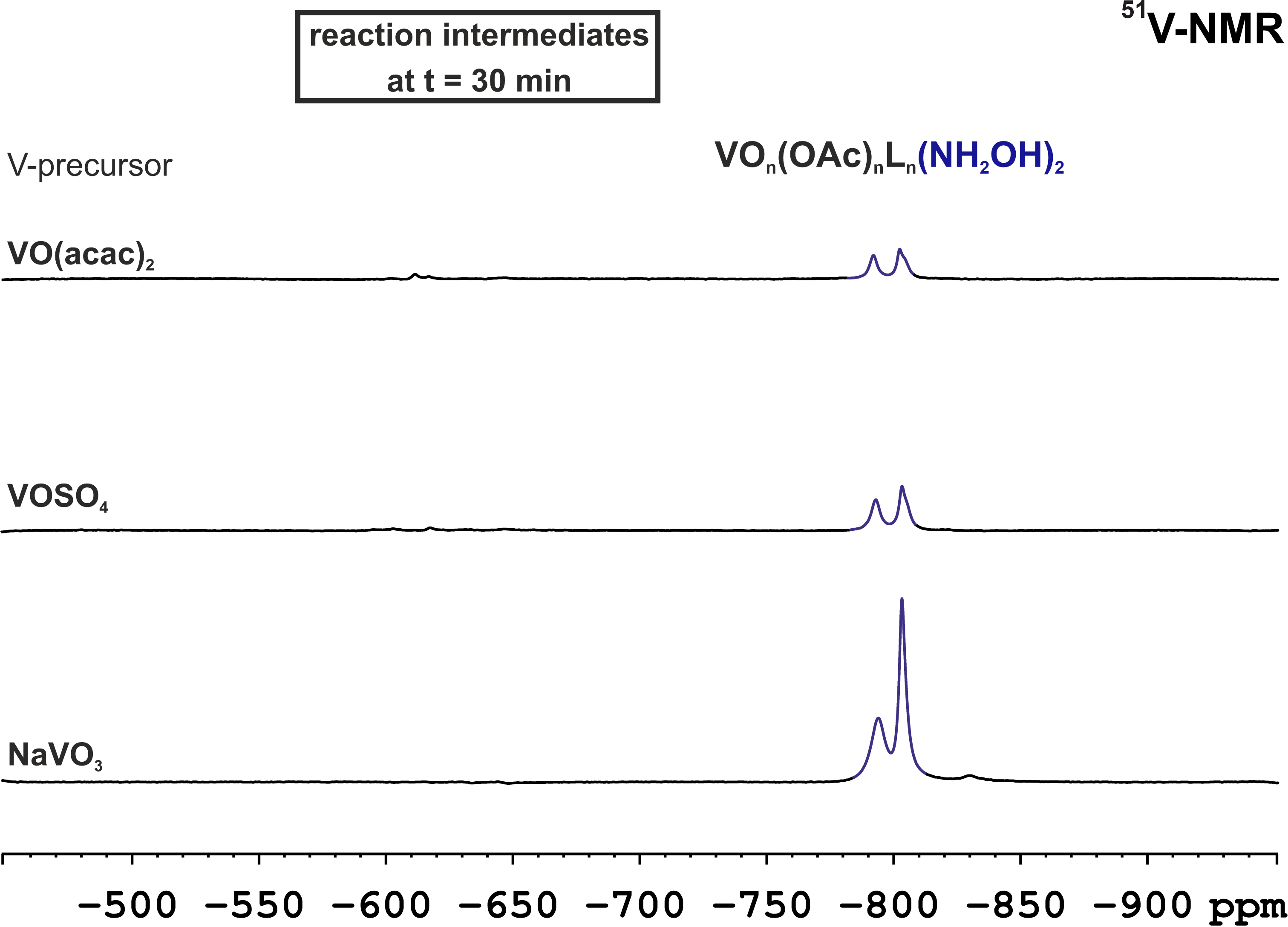
**

**Figure S14:** Ex situ vanadium reaction intermediates at t = 30 min derived from ^51^V-NMR of the ex situ photocatalytic amination of benzene with the vanadium catalysts VOSO_4_ and VO(acac)_2_ with the oxidation state of V^+IV^ and with the vanadium catalyst NaVO_3_ with the oxidation state of V^+V^. Reaction conditions: 0.5 mmol benzene, 5 equiv. NH_2_OH·HCl, 10 mol% V-catalyst, 1 mol% N-phenylphenothiazine, 1 mL 70% aq. AcOH, LED (365 nm).


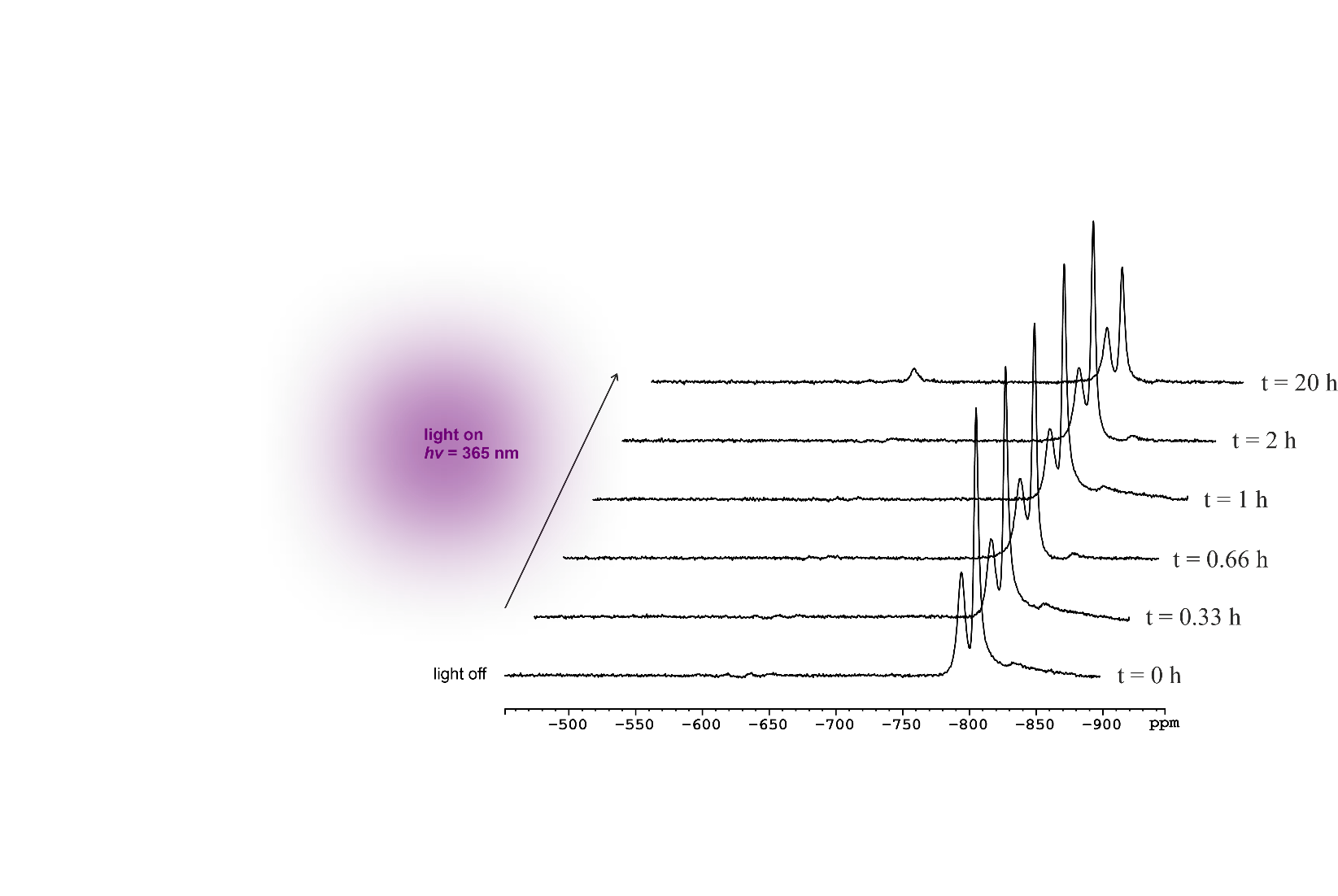


**Figure S15:** ^51^V-NMR spectra at different time points of the photocatalytic amination of benzene (0.5 M) with NaVO_3_ (10mol%) and PC (2 mol%) as catalysts and 5 equiv. NH_2_OH∙HCl in deuterated acetic acid/water 7:3. The V^V^ is always clearly present in its diamagnetic bis-hydroxylamine complexes throughout the whole reaction. Portions of the catalyst are reduced to V^IV^. Spectra are recorded after different time points. ^51^V-NMR: 600 MHz, 298K, pulsprog: msc_zg, NS: 4096.


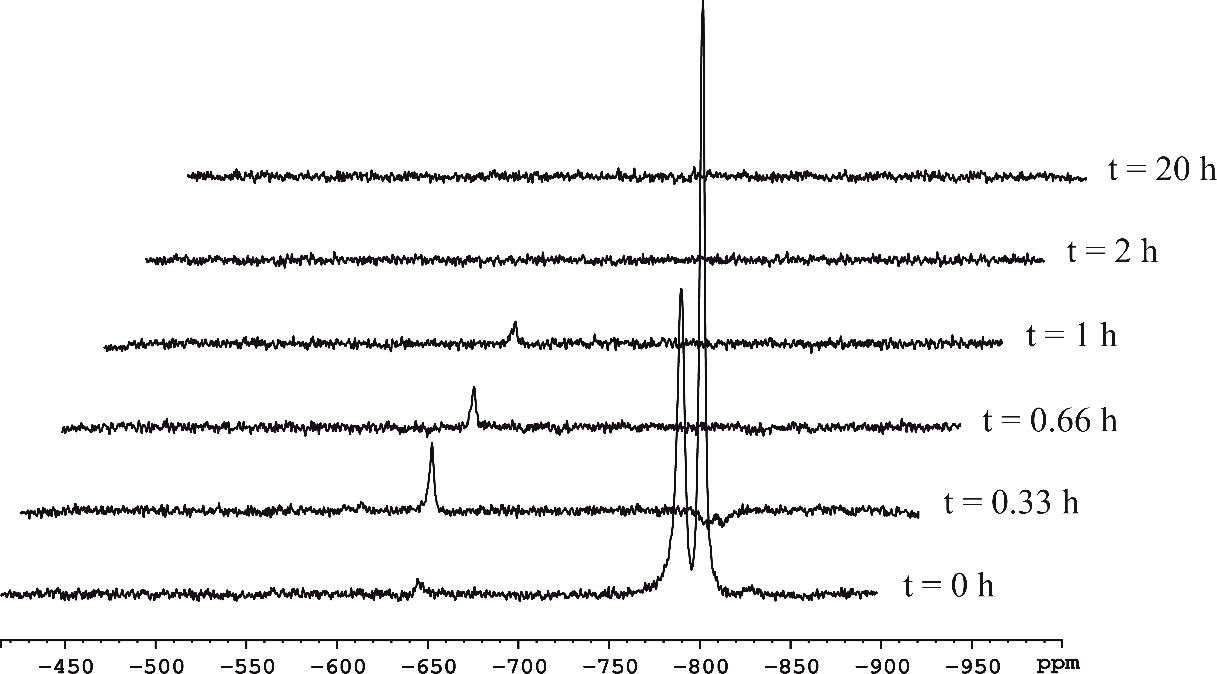


**Figure S16:** ^51^V-NMR spectra at different time points of the thermal amination of benzene (0.5 M) with NaVO_3_ (10 mol%) as catalysts and 5 equiv. NH_2_OH∙HCl in deuterated acetic acid/water 7:3. The V^V^ is reduced to V^IV^ within a few minutes of the reaction. Then mono-hydroxylamine vanadium complexes at around -650 ppm are observed. Spectra are recorded after different time points. ^51^V-NMR: 600 MHz, 348 K, pulsprog: zg, NS: 4096.

Pre-reduction of the vanadium(V) catalyst by stirring for 30 min in dark led to an increased reaction rate. After 30 min reaction time, bis-hydroxylamine vanadium species with resonances around −800 ppm were observed. In contrast, complete reduction of vanadium(V) to vanadium(IV) resulted in the absence of bis-hydroxylamine complexes, with only minor signals attributed to mono-hydroxylamine species remaining. Since vanadium(IV) species are paramagnetic and not directly observable by NMR, we hypothesise that prolonged pre-reduction generates kinetically inert or catalytically inhibiting V(IV) species, which also cannot undergo re-oxidation to vanadium(V) bis-hydroxylamine under the reaction conditions. These indicate that the presence of vanadium(V)-bis-hydroxylamine as an intermediate is a marker for efficient aniline formation, likely in dynamic equilibrium with catalytically active vanadium(IV)-hydroxylamine intermediates.


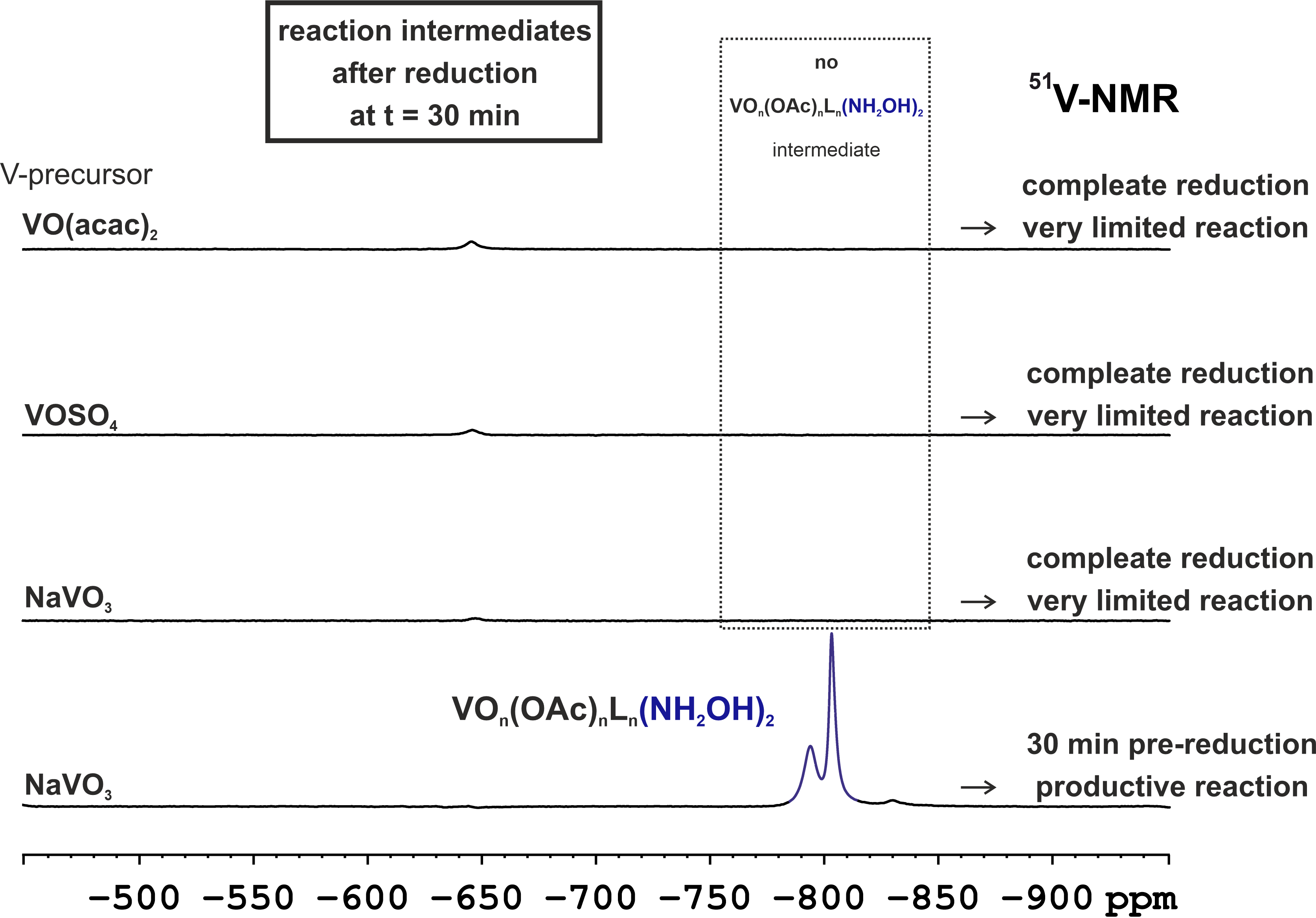


**Figure S17:** Ex situ vanadium reaction intermediates to compare partial reduction and full prereduction at t = 30 min derived from ^51^V-NMR of the ex situ photocatalytic amination of benzene with the vanadium catalysts VOSO_4_ and VO(acac)_2_ with the oxidation state of V^+IV^ and with the vanadium catalyst NaVO_3_ with the oxidation state of V^+V^. Full reduction of the catalysts for 6 h in the ultrasonic bath at 65 °C till a significant colour change appears and no V^+V^ visible in the ^51^V-NMR. Pre-reduction of the catalysts for 30 min stirring at rt. Reaction conditions: 0.5 mmol benzene, 5 equiv. NH_2_OH·HCl, 10 mol% V-catalyst, 1 mol% N-phenylphenothiazine, 1 mL 70% aq. AcOH, LED (365 nm).

To gain further insight into the role of the photocatalyst within the catalytic cycle, vanadium intermediates were monitored under irradiation in the presence and absence of the photocatalyst but without benzene as a substrate. Identical experimental conditions were used to allow a direct qualitative comparison. In the presence of the reducing PPT photocatalyst, a higher fraction of vanadium(V) species is observed compared to the control without photocatalyst. This finding can be explained by lacking the electron coming from the substrate re-aromatisation step, therefore, the cycle can be closed by oxidation of vanadium(IV) to vanadium(V). On the other hand, the mixture without photocatalyst stays in the vanadium(IV) oxidation state.

*
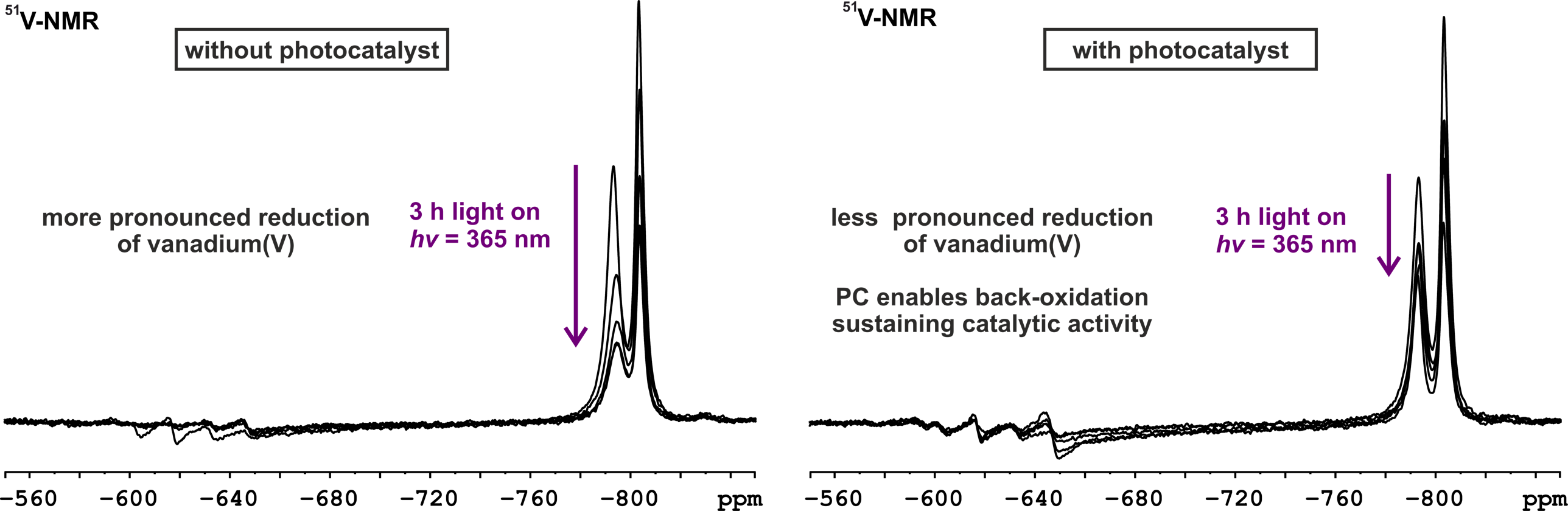
*

**Figure S18:** Ex situ vanadium(V) hydroxylamine of the illumination for 3 h without substrate to assess the potential role of the photocatalyst. Compared are the vanadium species in the presence (right spectra) and absence (left spectra) of the photocatalyst. Prolonged illumination leads to reduction in both cases with varying degrees. With PC more vanadium species is present in the oxidated vanadium(V) bis-hydroxylamine form. Reaction conditions: 2.5 M NH_2_OH·HCl, 0.05 M NaVO_3_, 0.005 M N-phenylphenothiazine (right) **or** no PC (left), 1 mL 70% aq. AcOH, LED (365 nm).

**^15^N-NMR kinetics**

The ^15^N-NMR kinetics reveal the formation of aniline, N_2_O, and ammonium ions, with no additional nitrogen-containing side products detected in the spectra.


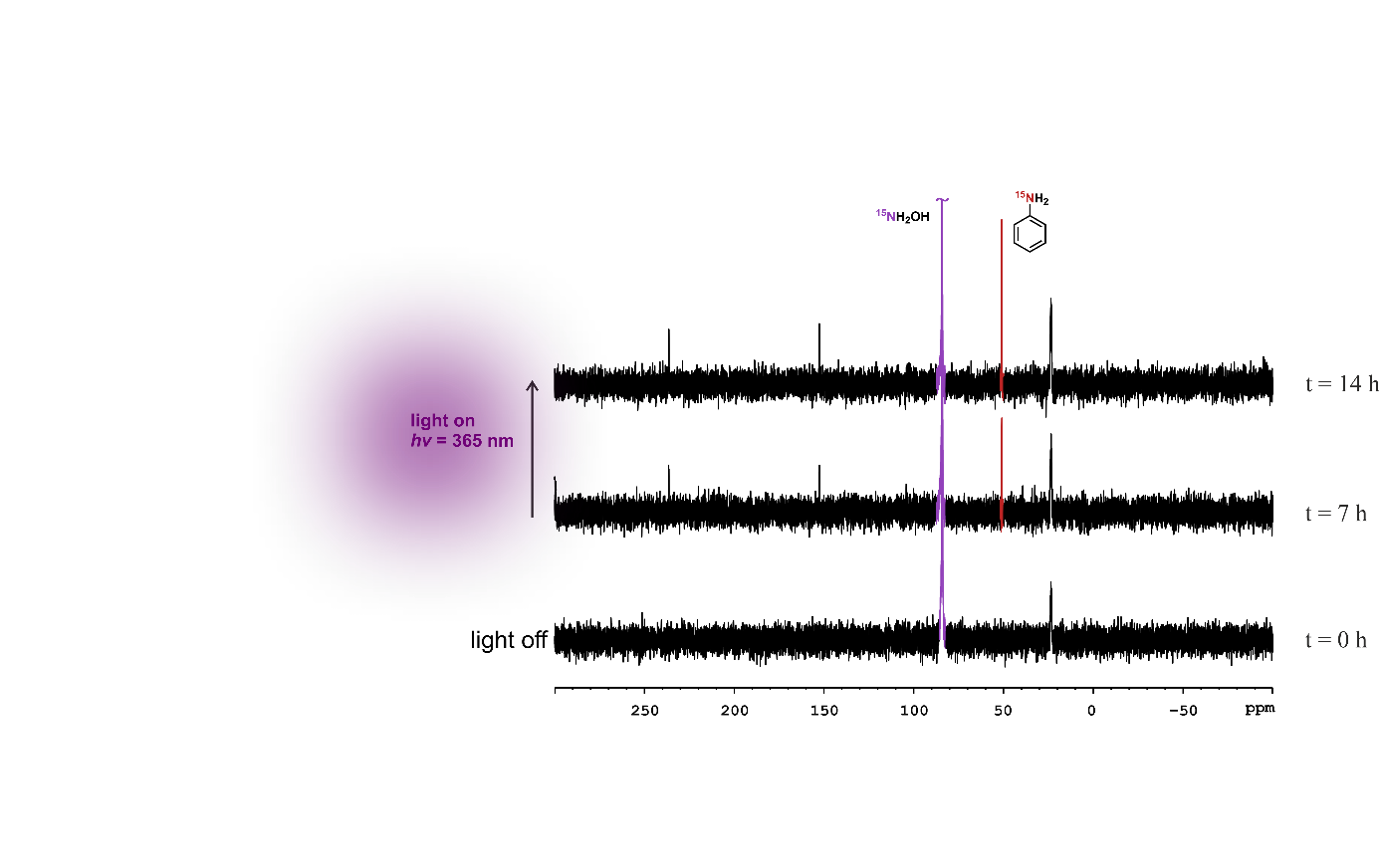


**Figure S19:** ^15^N-NMR spectra at different time points of the photocatalytic amination of benzene (0.5 M) with 5mol% NaVO_3_ 5mol% PC and 5 equiv. of ^15^NH_2_OH∙HCl in deuterated acetic acid/water 7:3. Clearly the formation of ^15^N‑aniline, N_2_O and the proposed V^IV^ hydroxylamine catalyst is visible. A) First spectrum before the start of the reaction. b) Second spectrum after 7 h reaction time. c) Third spectrum after 14 h reaction time.

**^15^N-NMR for Structural Insights into Paramagnetic Vanadium Complexes**

Building on the previous EPR data, the immediate partial reduction of NaVO_3_ catalyst by hydroxylamine in an acidic medium is evident (**Figure 3**). This time-dependent reduction transforms V^+5^ to V^+4^, making it undetectable by ^51^V-NMR due to its paramagnetic nature. To overcome this limitation, ^15^N-labelled hydroxylamine could provide valuable insights into the system. In contrast to ^51^V-NMR, the ^15^N-NMR spectrum recorded after five days in strongly acidic conditions (pH ≤ ‑0.5) showed new peaks as V^+V^ was reduced to V^+4^, accompanied by decreased signal intensity and gradual vanishing of the signal (**Figure S19**). The initial ^15^N-NMR spectrum showed a peak at 83.9 ppm, assigned to free hydroxylamine (**Figure S7**), and a minor unassigned peak at 103.8 ppm. After 7 days, three new signals appeared at 236.5, 152.6, and 23.1 ppm (**Figure S24**). The first two peaks appeared as downfield-shifted doublets with J = 8.3 Hz, consistent with ^1^J_N-N_ coupling, and were assigned to N_2_O formed during the hydroxylamine-mediated reduction of V^+5^ to V^+4^.^[7]^


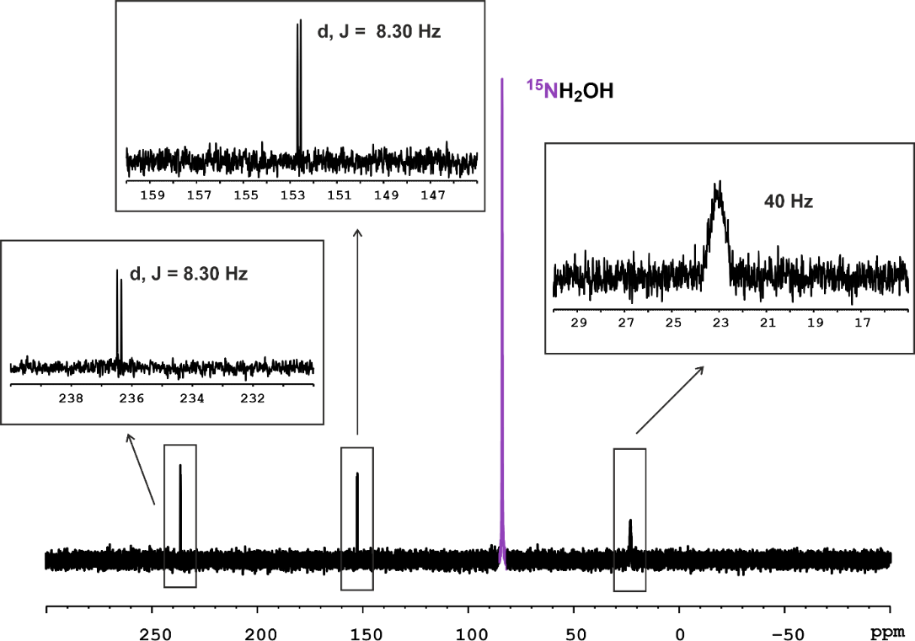


***Figure S20:*** *^15^N-NMR spectrum of the reduced NaVO_3_ catalyst (0.05 M) with 5 equiv. ^15^NH_2_OH∙HCl in HOAc/D_2_O (7:3). All peaks for the degradation products are displayed in the zoomed windows. The two peaks with a doublet each at around 235 ppm and 150 ppm resemble N_2_O and the broadened peak at around 20 ppm NH_4_^+^.*

The peak at 23.1 ppm broadened to approximately 40 Hz due to exchange processes. Its assignment as free ammonium ion was confirmed by titration with ^15^N-labelled ^15^NH_4_Cl and reflects decomposition of hydroxylamine over time (**Figures S21, S22**). Because V^+4^ is paramagnetic and causes NMR signals of nearby nuclei (up to ⁓9Å, lit. ^[8]^) invisible, no signal of V^+4^ complexes were observed. Titration with ^15^NH_4_Cl led to increased signal intensity in both the ^1^H- and ^15^N-NMR spectra of the previously unassigned species, confirming it as the ammonium-ion NH^4+^. The observed doublet originates from scalar coupling to ¹⁵N (I = ½) in a symmetric environment. In contrast, ^1^H-NMR of ^14^N‑hydroxylamine shows a triplet, consistent with coupling to ^14^N (I = 1).


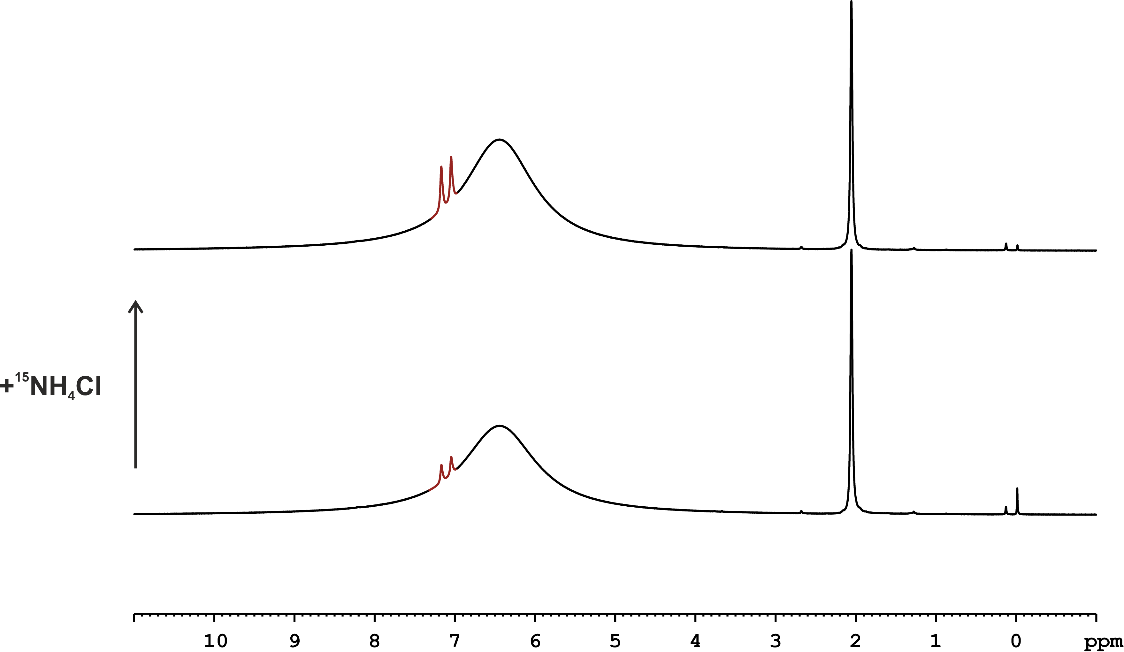


**Figure S21:** ^1^H-NMR spectra of an already reduced sample (over a week old) of NaVO_3_ catalyst (0.05 M) with 50 eq of ^15^N-labled hydroxylamine in acetic acid/D_2_O 7:3 to investigate the unknown doublet at 7.1 ppm. Lower spectrum: no deviation of the conditions. Prior reduction of the sample shows a signal (doublet) at 7.1 ppm. Upper spectrum: addition of ^15^NH_4_Cl leads to an increase in the respective signal. 600 MHz, 298K.


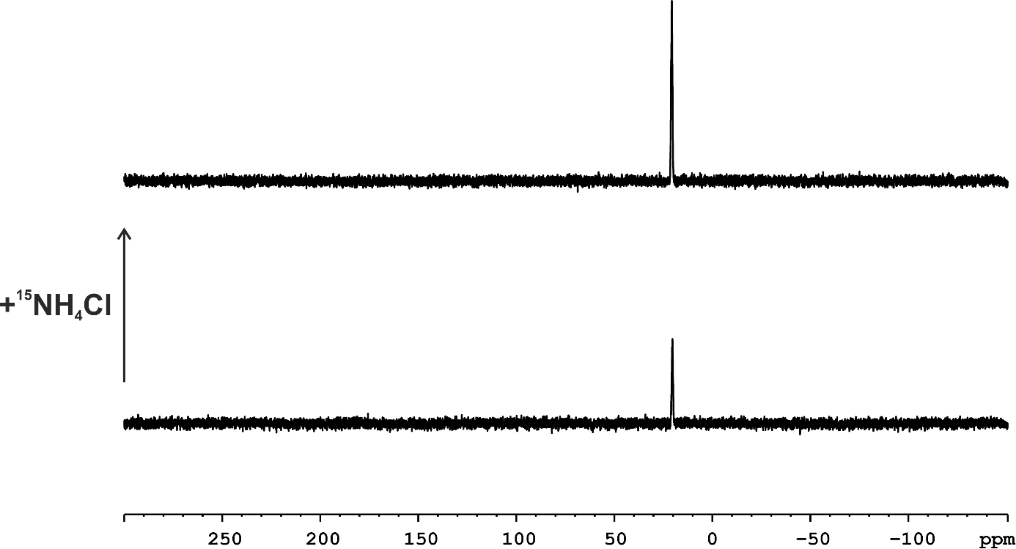


**Figure S22:** ^15^N-NMR spectra of an already reduced sample (over a week old) of NaVO_3_ catalyst (0.05 M) with 50 eq of ^15^N-labled hydroxylamine in acetic acid/D_2_O 7:3 to investigate the unknown peak at around 20 ppm. lower spectrum: no deviation of the conditions. Prior reduction of the sample shows a signal at around 20 ppm. upper spectrum: addition of ^15^NH_4_Cl leads to an increase in the respective signal. 600 MHz, 298K, pulsprog: zgig30 (^1^H-decoupled), NS: 512.

Upon addition of hydroxylamine, the ^15^N-NMR signal of aniline sharpens. This behaviour may indicate interactions between aniline and vanadium complexes, potentially contributing to the observed product inhibition.


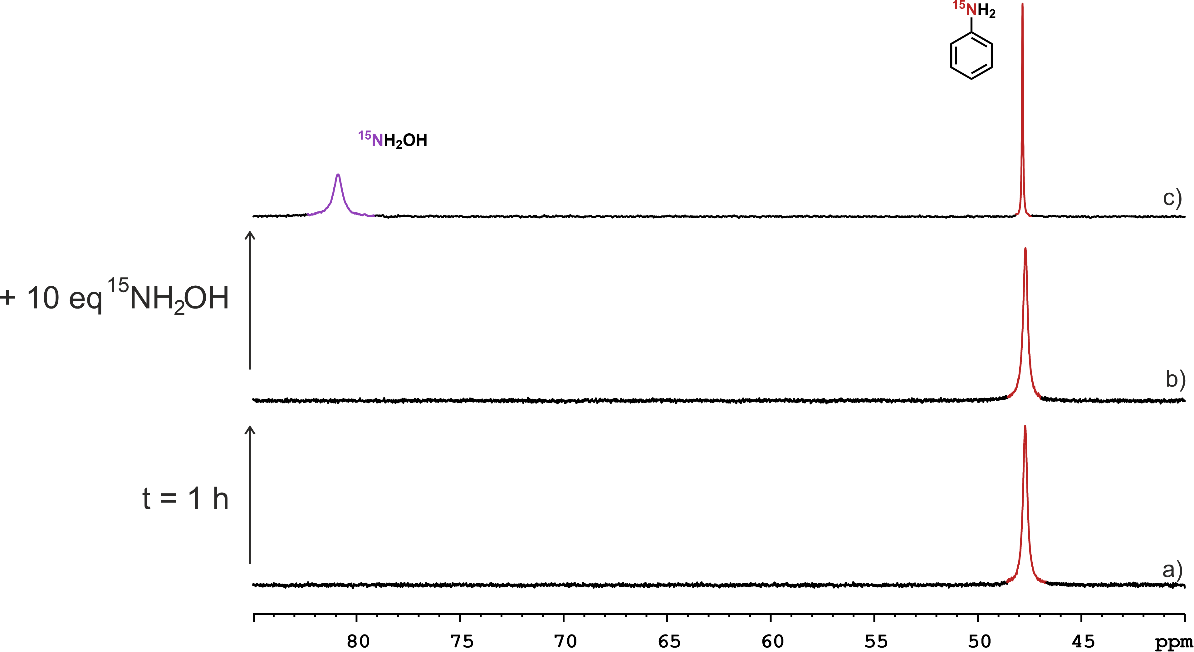


**Figure S23:** ^15^N-NMR spectra of 10 equiv. ^15^N-labled aniline with NaVO_3_ catalyst (0.05 M) in deuterated acetic acid/water 7:3 to investigate the aniline interactions. a: no deviation of the conditions. b: waiting for 1 h leads to a vaguely further broadening of the aniline. c: addition of 10 equiv. of ^15^NH_2_OH∙HCl. This results in a narrowing of the peak for the aniline species. 600 MHz, 298K, pulsprog: zgpg30 (^1^H-decoupled), NS: 512.


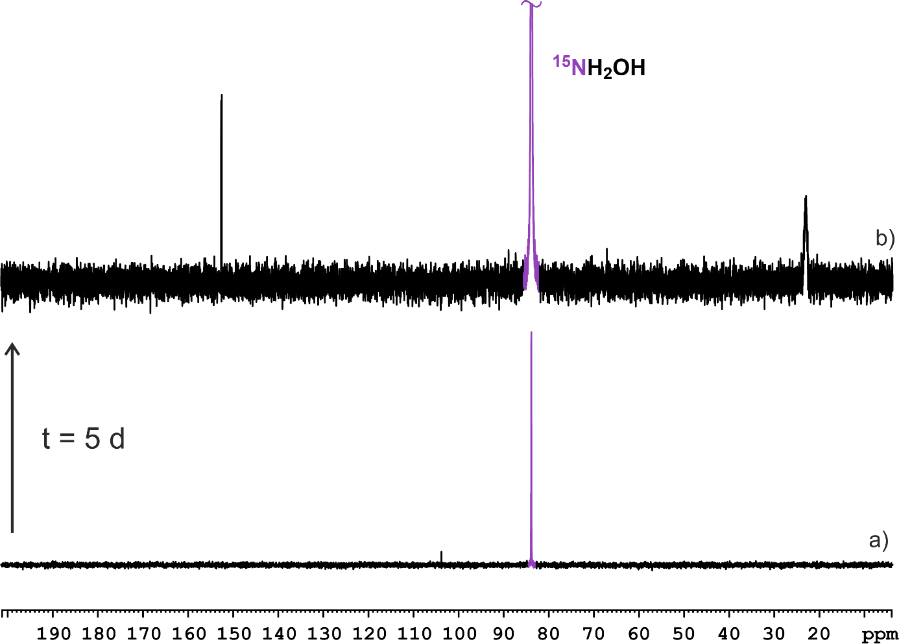


7 days

***Figure S24****: ^15^N-NMR spectrum of NaVO_3_ (0.05 M) with 5 equiv. ^15^NH_2_OH∙HCl in DOAc-d_4_/D_2_O 7/3. a: First spectrum directly after preparation of the sample. b: Second spectrum after 7 days. Upon reduction additional nitrogen-containing species are visible. pulseprog: zgpg30, NS: 512.*


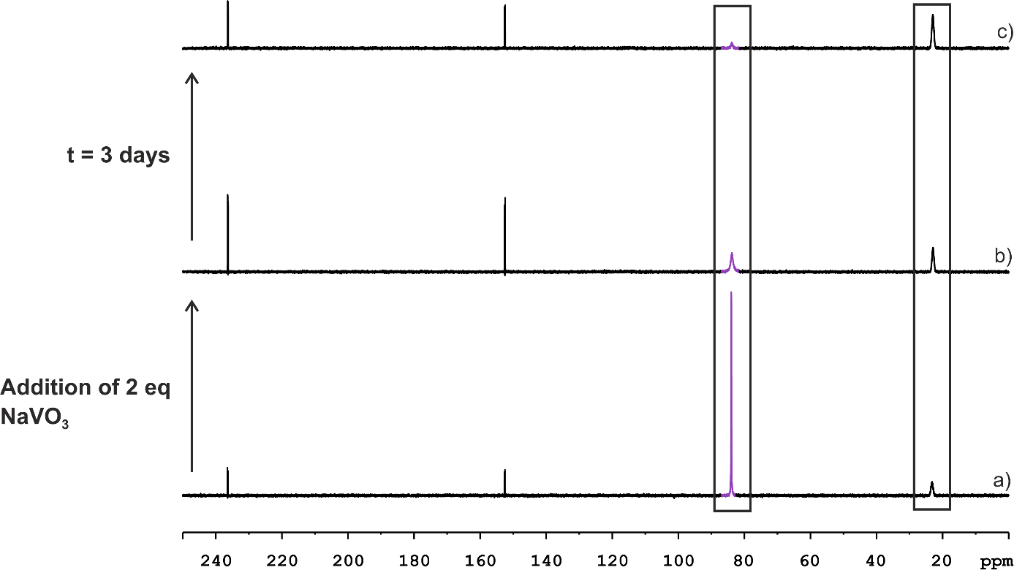


***Figure S25****: ^15^N-NMR spectrum of reduced NaVO_3_ (0.05 M) in DOAc-d_4_/D_2_O 7:3 with 5 equiv. of ^15^N-labeled Hydroxylamine. a) First spectrum without any deviation. b) Second spectrum after addition of 2 equiv. NaVO_3_. c) Third spectrum after 3 days. pulseprog: zgpg30, NS: 2048. Zoomed in region shows the increase of NH_4_^+^ peak at 23 ppm.*


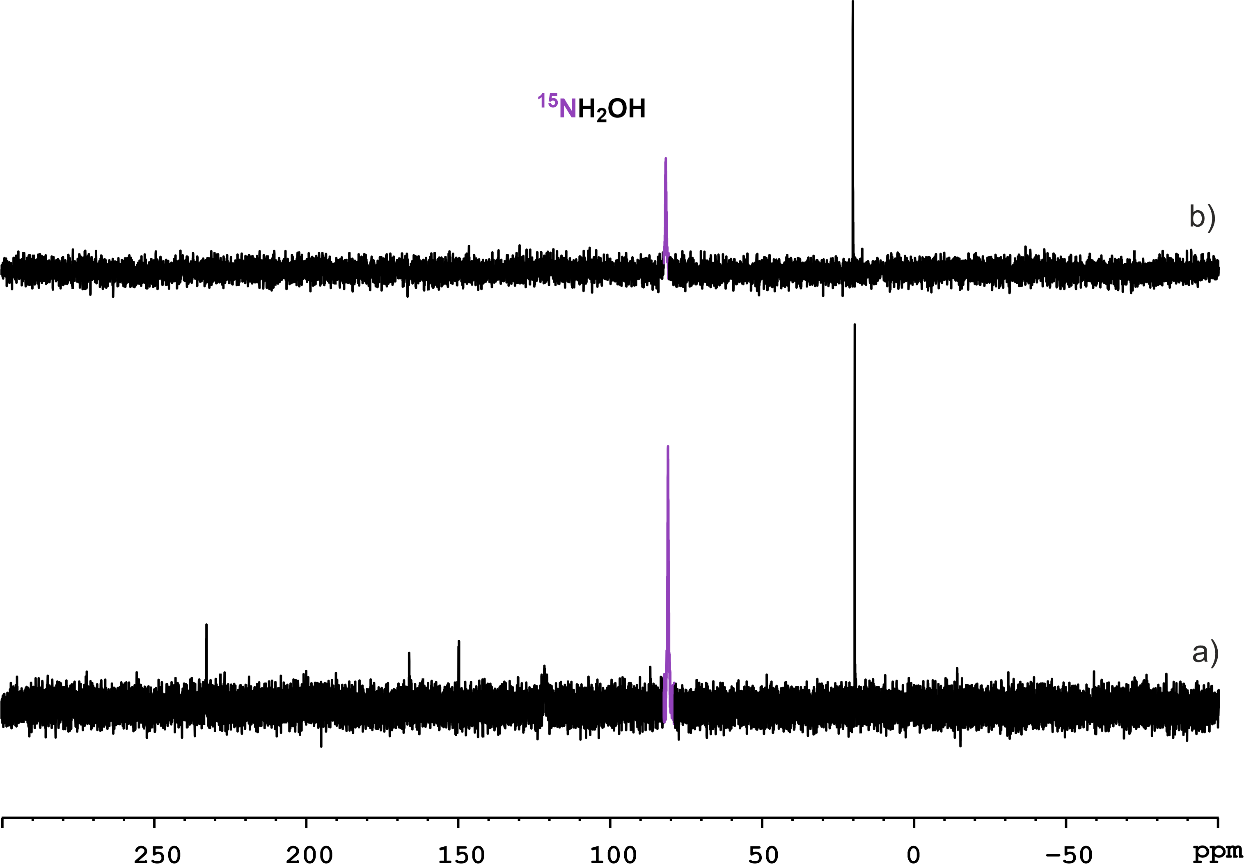


**Figure S26:** ^15^N-NMR spectrum of NaVO_3_ (0.025 M) with 10 equivalents ^15^NH_2_OH∙HCl in DOAc-d_4_/D_2_O 3.5:6.5 with an addition of 600 mg KOAc (1 equiv. regarding to the acetic acid) to create a DOAc/KOAc buffer system with an elevated pH to the very low pH of the conditions (pH≈6). A) no variation of the noted conditions. b) addition of 0.1 mL NaOH to lower the pH to basic conditions (pH≈8). 600 MHz, 298K, pulsprog: zgpg30 (^1^H-decoupled), NS: 512.

The absence of N_2_O and NH_4_^+^ formation upon addition and presence of VO(acac)_2_, a vanadium(IV) species that cannot be further reduced by hydroxylamine, demonstrates that these nitrogen products originate from the reduction of vanadium(V)–hydroxylamine complexes to vanadium(IV)–hydroxylamine species. (**Figure S27**)


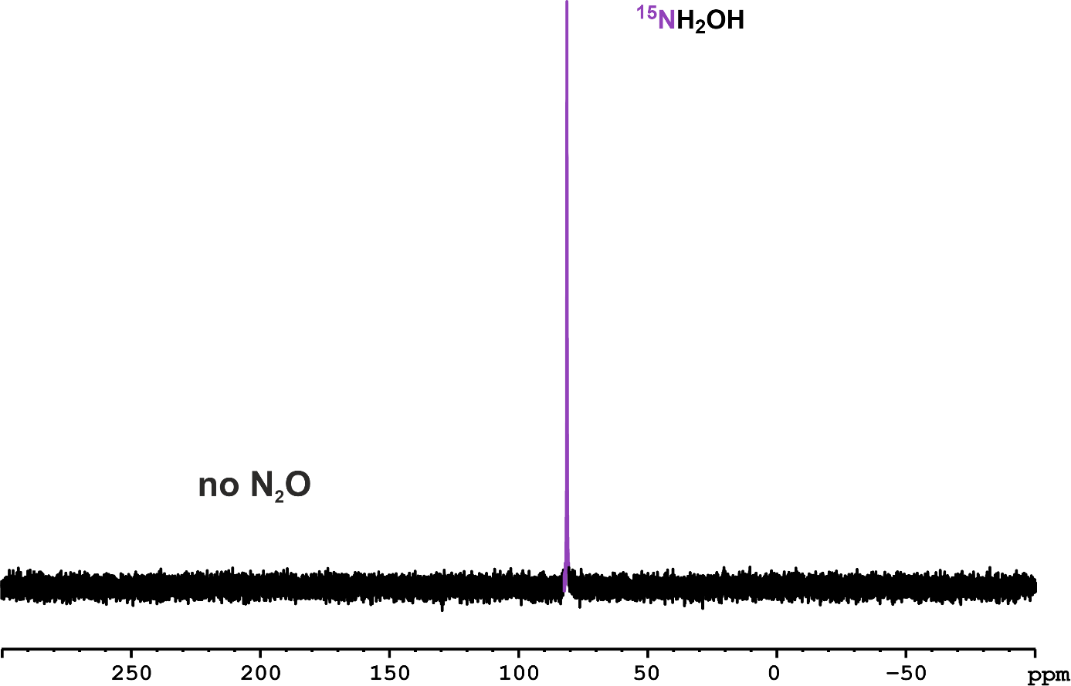


**Figure S27:** ^15^N-NMR spectrum of 0.05 M VO(acac)_2_ with ⁓ 1 equivalent ^15^NH_2_OH∙HCl in HOAc/D_2_O 3:7. No hydroxylamine degradation product is formed after 3 days. After addition of more VO(acac)_2_ catalyst still no degradation product is formed. 600 MHz, 298K, pulsprog: zgpg30 (^1^H-decoupled), NS: 1024.


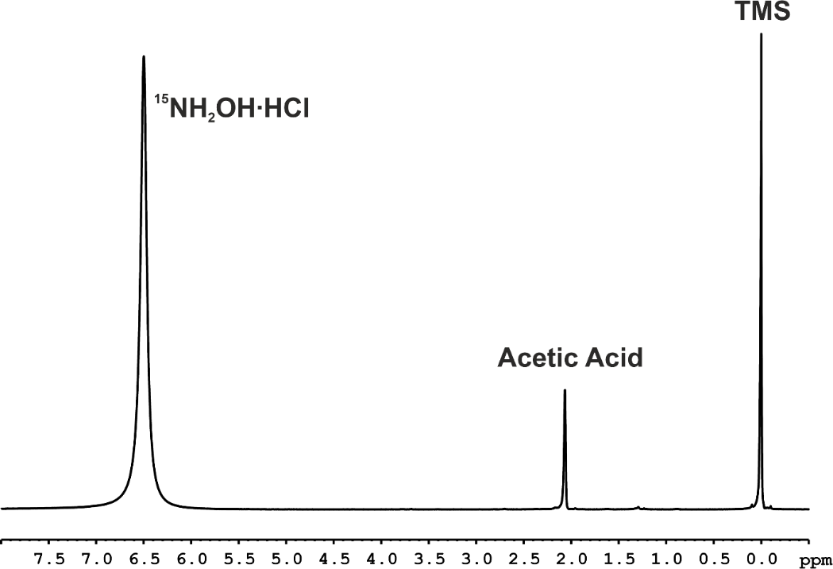


**Figure S28:** ^1^H-NMR spectra of ^15^NH_2_OH∙HCl in D_2_O/DOAc-d_4_. Without vanadium species, no additional H-bond is shown, and the proton peaks of hydroxylamine are not as severely broadened. ^1^H-NMR: 600 MHz, 298K, pulsprog: zg, NS: 4.

# EPR spectroscopy


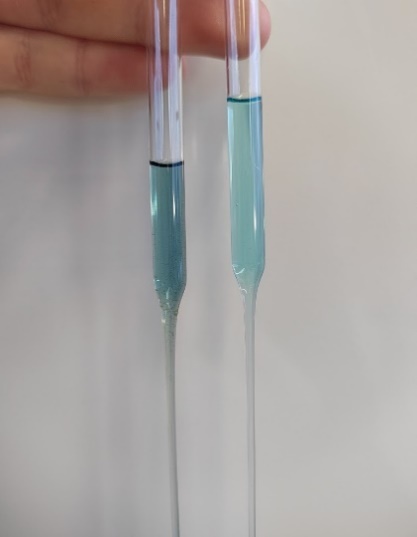

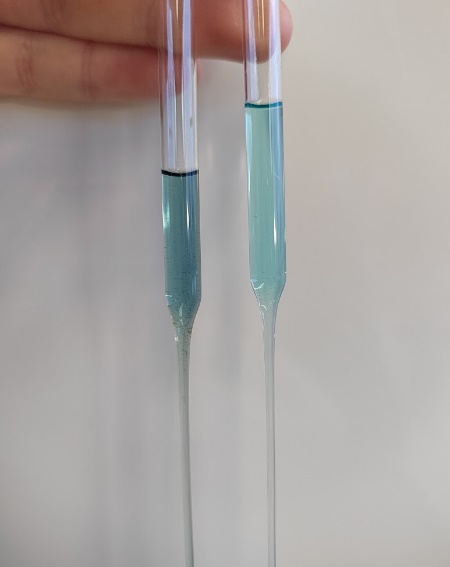


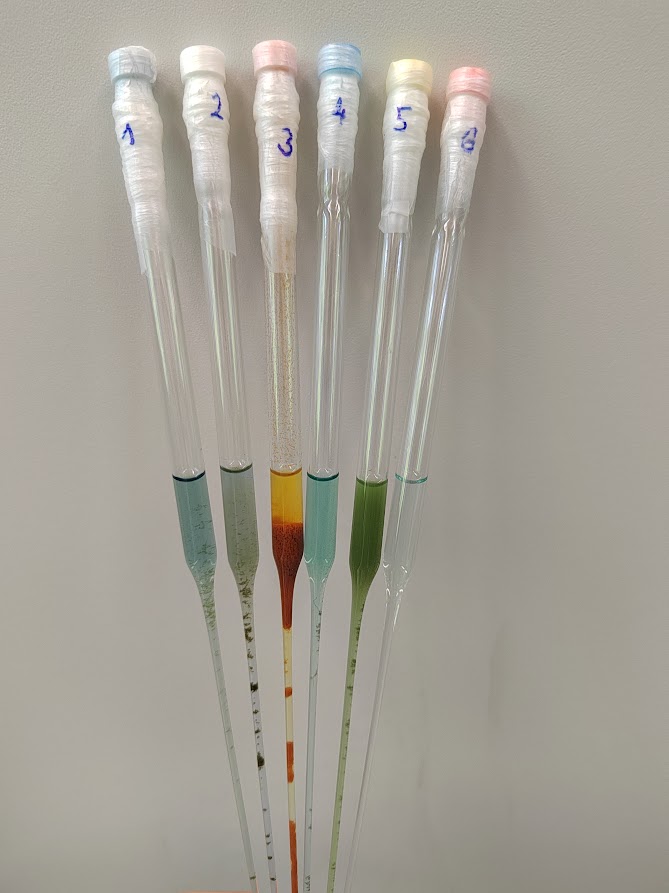

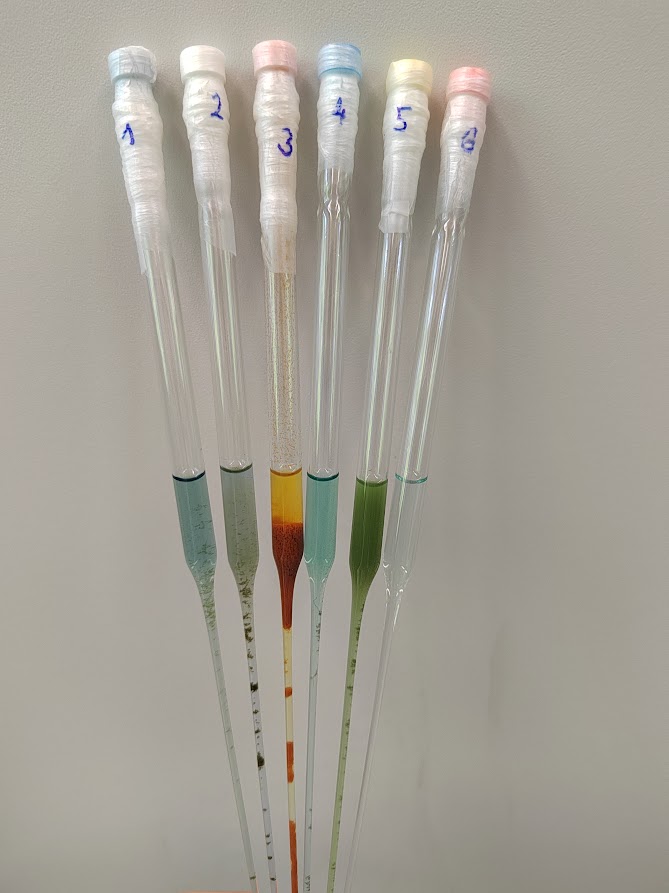


***Figure S29****:* ***A****: VO(acac)_2_, ν_mw_= 9.458835 GHz.* ***B****: VO(acac)_2_ + NH_2_OH·HCl, ν_mw_= 9.459400 GHz.* ***C****: VO(OAc)_2_, ν_mw_= 9.458626 GHz.* ***D****: VO(OAc)_2_ + NH_2_OH·HCl, ν_mw_= 9.458893 GHz.*

# Effect of LED radiant power and GC analysis

The reaction progress is not faster only at higher temperatures, but also the higher radiant power of LEDs rapidly speeds up the aniline formation (**Figure S30**). In the case of the reaction with the VO(acac)_2_ catalyst, the aniline yield of 45%, which is reached after 16 h of irradiation by standard-power LEDs (~500 mW), was gained within 1 hour of irradiation with high-power LEDs (~3 W). In addition, the higher radiant power increases the overall yield by 10%, which could be caused by local heating.

**Figure S30:** Aniline formation with 10 mol% of VO(acac)_2_ at 40 °C with high and standard radiant power LEDs.


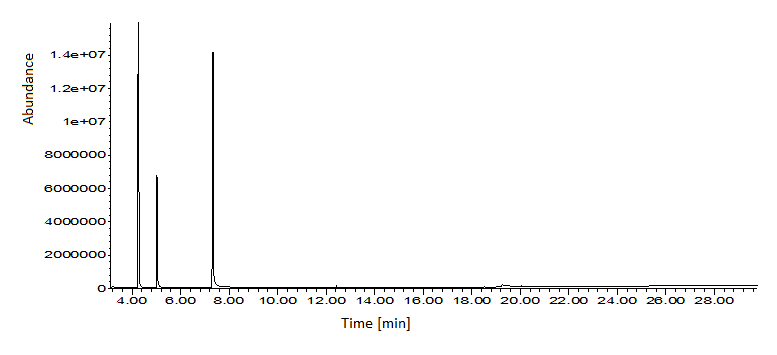


**Figure S31:** GC chromatogram of the reaction mixture with VO(acac)_2_ and the mass spectrum of the signal at 5.035 min (red) compared with the corresponding library spectrum of 3,5-dimethylisoxazole (blue).


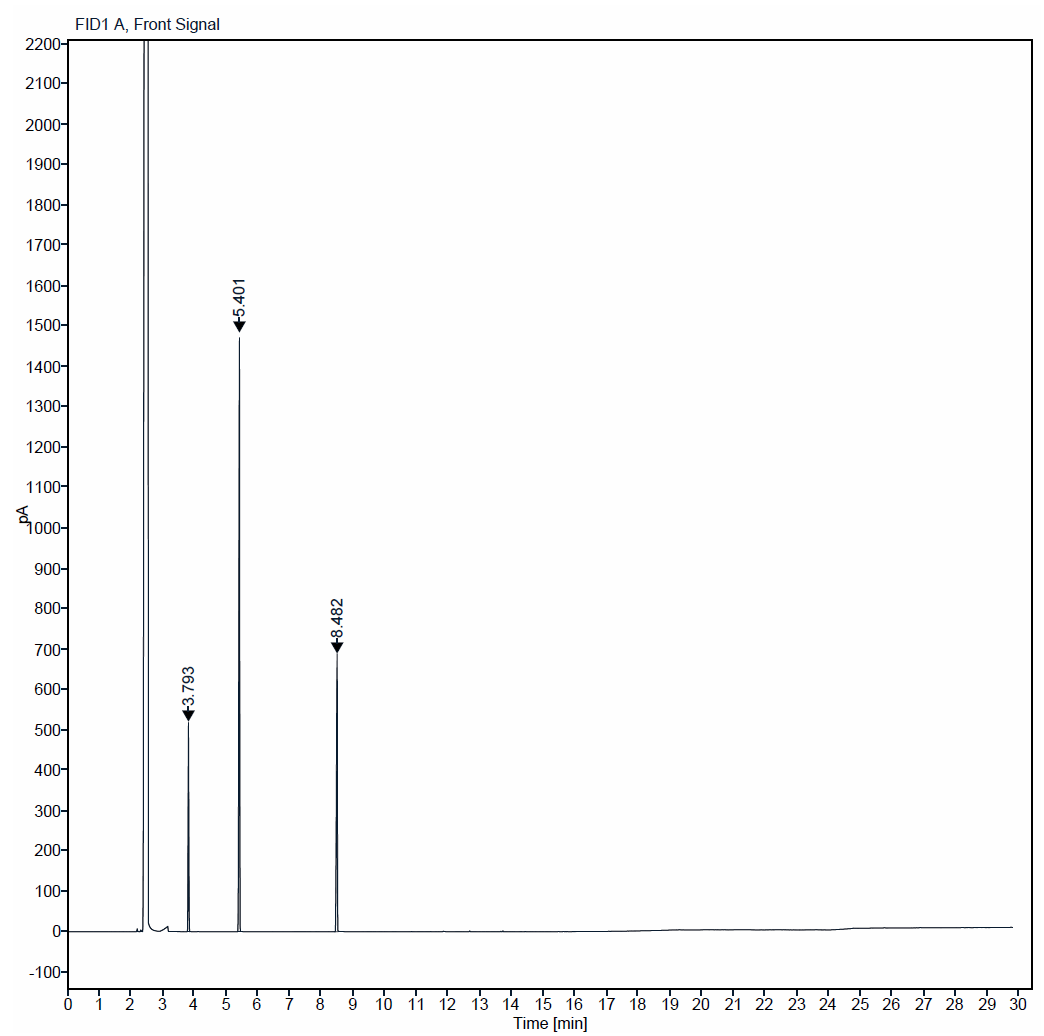


***Figure S32:*** *GC-FID chromatogram of the reaction mixture with VO(OAc)_2_ after 4 h of irradiation, neutralisation, extraction with Et_2_O, and addition of toluene as an internal standard. RT_benzene_ = 3.8 min; RT_toluene_ = 5.4 min; RT_aniline_ = 8.5 min.*

# References

[1] C. Feldmeier, H. Bartling, E. Riedle, R. M. Gschwind, *J. Magn. Reson.* **2013**, *232*, 39-44.

[2] R. K. Harris, E. D. Becker, S. M. C. d. Menezes, R. Goodfellow, P. Granger, *Pure Appl. Chem.* **2001**, *73*, 1795-1818.

[3] L. F. Zhu, B. Guo, D. Y. Tang, X. K. Hu, G. Y. Li, C. W. Hu, *J. Catal.* **2007**, *245*, 446-455.

[4] R. P. Brooker, C. J. Bell, L. J. Bonville, H. R. Kunz, J. M. Fenton, *J. Electrochem. Soc.* **2015**, *162*, A608.

[5] D. Rehder, *Coord. Chem. Rev.* **2008**, *252*, 2209-2223.

[6] P. Nitschke, N. Lokesh, R. M. Gschwind, *Prog. Nucl. Magn. Reson. Spectrosc.* **2019**, *114-115*, 86-134.

[7] H. Schultheiss, E. Fluck, *Z. Naturforsch. B* **1977**, *32*, 257-264.

[8] E. Ravera, L. Gigli, L. Fiorucci, C. Luchinat, G. Parigi, *Phys. Chem. Chem. Phys.* **2022**, *24*, 17397-17416.
